# Glucagon-like peptide-1 receptor agonists for prevention of heart failure events in type 2 diabetes and/or obesity

**DOI:** 10.1093/eschf/xvag091

**Published:** 2026-03-25

**Authors:** Keerthenan Raveendra, Tobin Joseph, Jing Kang, Ben Hurdus, Uther Cutting, Mohammad Haris, Tanina Younsi, Amaan Aslam, Mark Petrie, Marco Metra, Ramzi A Ajjan, Jianhua Wu, Chris P Gale, Ramesh Nadarajah

**Affiliations:** Faculty of Medicine and Health, University of Leeds, Leeds, UK; Leeds Institute for Cardiovascular and Metabolic Medicine, University of Leeds, 6 Clarendon Way, Leeds LS2 9DA, UK; Centre for Clinical Translational Science, Faculty of Dentistry, Oral and Craniofacial Sciences, King’s College London, London, UK; Leeds Institute for Cardiovascular and Metabolic Medicine, University of Leeds, 6 Clarendon Way, Leeds LS2 9DA, UK; Faculty of Medicine and Health, University of Leeds, Leeds, UK; Leeds Institute for Cardiovascular and Metabolic Medicine, University of Leeds, 6 Clarendon Way, Leeds LS2 9DA, UK; Department of Cardiology, Leeds Teaching Hospitals NHS Trust, Leeds LS1 3AX, UK; Faculty of Medicine and Health, University of Leeds, Leeds, UK; Institute of Cardiovascular and Medical Sciences, University of Glasgow, Glasgow, UK; Institute of Cardiology, ASST Spedali Civili di Brescia and Department of Medical and Surgical Specialties, Radiological Sciences, and Public Health, University of Brescia, Brescia, Italy; Leeds Institute for Cardiovascular and Metabolic Medicine, University of Leeds, 6 Clarendon Way, Leeds LS2 9DA, UK; Wolfson Institute of Population Health, Queen Mary University of London, London, UK; Leeds Institute for Cardiovascular and Metabolic Medicine, University of Leeds, 6 Clarendon Way, Leeds LS2 9DA, UK; Department of Cardiology, Leeds Teaching Hospitals NHS Trust, Leeds LS1 3AX, UK; Leeds Institute of Data Analytics, University of Leeds, Leeds LS2 9NL, UK; Leeds Institute for Cardiovascular and Metabolic Medicine, University of Leeds, 6 Clarendon Way, Leeds LS2 9DA, UK; Department of Cardiology, Leeds Teaching Hospitals NHS Trust, Leeds LS1 3AX, UK; Leeds Institute of Data Analytics, University of Leeds, Leeds LS2 9NL, UK

**Keywords:** Heart failure, GLP-1 receptor agonists, Type 2 diabetes, Obesity, Meta-analysis

## Abstract

**Introduction:**

Heart failure (HF) is a common sequela of diabetes and obesity. Glucagon-like peptide-1 (GLP-1) receptor agonists may prevent HF events. This meta-analysis estimates absolute risk reduction (ARR) and number needed to treat (NNT) for GLP-1 receptor agonists to prevent one HF event in patients with type 2 diabetes and/or obesity, including those without baseline HF.

**Methods:**

The Medline, Embase, and Cochrane Central databases were searched to 04 April 2025 for placebo-controlled randomized controlled trials (RCTs) of GLP-1 receptor agonists in a type 2 diabetes and/or obesity indication with a prespecified HF event endpoint. Random effects meta-analysis using the Mantel-Haenszel Method was performed to synthesize risk ratios (RR), ARRs, and NNTs with 95% confidence intervals (CI).

**Results:**

Twelve placebo-controlled RCTs involving 95 023 patients were included. GLP-1 receptor agonists reduced HF events by 12% (RR 0.88, 95% CI 0.82–0.95; ARR 0.42%, 95% CI 0.17%–0.62%; NNT 238, 95% CI 161–588), and, in those without baseline HF, by 19% (RR 0.81, 95% CI 0.72–0.90; ARR 0.60%, 95% CI 0.32%-0.89%; NNT 167, 95% CI 113–313). Semaglutide reduced the risk of HF events by 16% (RR 0.84, 95% CI 0.74–0.95; ARR 0.62%, 95% CI 0.19%-1.00%; NNT 161, 95% CI 100–526), and by 31% in those without baseline HF (RR 0.69, 95% CI 0.55–0.88; ARR 1.25%, 95% CI 0.48%-1.82%; NNT 80, 95% CI 55–208).

**Conclusion:**

GLP-1 receptor agonists have limited absolute benefit for preventing HF events in patients with type 2 diabetes and/or obesity, including in those without baseline HF.

**Systematic review registration:**

PROSPERO: CRD420251074882

## Graphical Abstract

**Graphical Abstract:**
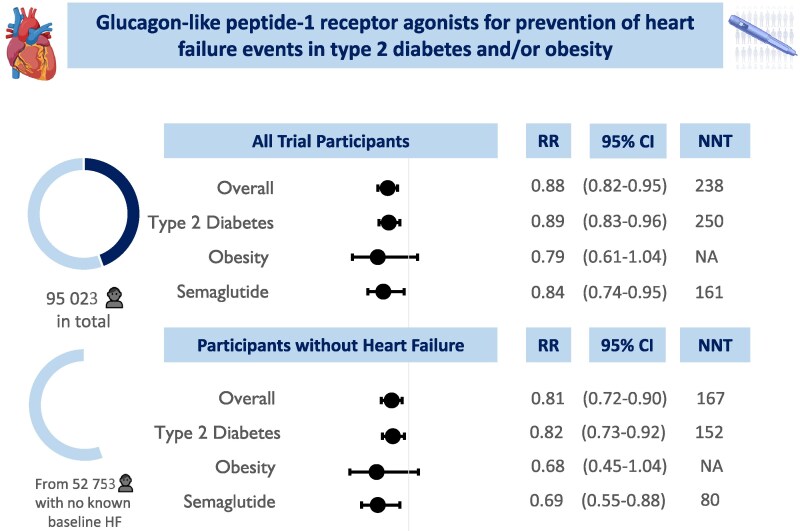
GLP-1 receptor agonists reduce heart failure events in patients with type 2 diabetes and/or obesity, including in those without baseline heart failure. CI, confidence interval; RR, risk ratio.

## Introduction

Glucagon-like peptide-1 (GLP-1) receptor agonists have been studied in several large cardiovascular outcomes trials in patients with type 2 diabetes mellitus (diabetes) and/or obesity. The risk of heart failure (HF) events (hospitalization or urgent clinical visit) is elevated in patients with diabetes, obesity, or both.^1^ HF events contribute the majority of costs for HF care,^2^ and patients with diabetes and/or obesity have worse outcomes after an HF event than those without diabetes and/or obesity.^3,4^

Trials of GLP-1 receptor agonists in patients with diabetes and/or obesity have been powered for a primary composite outcome of cardiovascular death, non-fatal myocardial infarction, and non-fatal stroke, but did not include HF. Recent meta-analyses attempted to circumvent this issue, and their findings suggest that GLP-1 receptor agonists reduce HF events in patients with diabetes and/or obesity, and that effects are pronounced in those without baseline HF.^5,6^ However, these analyses did not estimate the magnitude of absolute risk reduction (ARR) and thus the number needed to treat (NNT), which are important to patients, clinicians, payers, and policymakers.

The goal of the present meta-analysis was to provide contemporary, comprehensive, and clinically relevant risk estimates from placebo-controlled randomized controlled trials (RCTs) of GLP-1 receptor agonists to better assess their effectiveness for preventing HF events in patients with diabetes and/or obesity, including in those without known HF at baseline.

## Methods

This study was registered on PROSPERO (CRD420251074882) and informed by the PRISMA statement.^7^

### Data sources and searches

We conducted a systematic search of the Medline, Embase, and Cochrane Central databases from inception to 04 April 2025. A qualified research librarian contributed to the development of the search strategy. We used a combination of keywords and subject headings related to HF and GLP-1 receptor agonists based on previous literature (Supplementary Sections S1–S3).^8,9^ We completed forward and backward citation searching for included studies. Duplicates were removed using EndNote, and then checked manually.

### Study selection criteria

We included placebo-controlled RCTs of GLP-1 receptor agonists where prevalent HF was not part of the eligibility criteria, HF events were a primary or secondary endpoint, and HF events were reported in a published analysis.

### Data extraction and quality assessment

Three investigators (KR, UC, TJ) independently screened articles for inclusion by title, abstract, full text, and supplemental materials. Disagreements were resolved by consultation with another investigator (RN). Six investigators (KR, UC, TJ, AA, BH, HM) independently extracted information from the included studies, including that pertaining to the intervention and outcome, and study and participant characteristics. We extracted the number of HF events in the intervention and control groups, specifically for patients without baseline HF when such stratified analyses were reported. We also extracted the number of events in the intervention and control groups for safety outcomes frequently reported (pancreatitis, severe hypoglycaemia, acute renal failure, and cancer). Disagreements about data were discussed with two other investigators (JW and JK). Two investigators (KR and AA) assessed risk of bias using the modified Cochrane Collaboration’s risk of bias tool.^10^

### Statistical analysis

We calculated unadjusted risk ratios (RR) with 95% confidence intervals (CI) for each study to report effect estimates for HF events. We performed random effects meta-analysis to synthesize the overall RR, and by subgroup of indication, and present forest plots. ARR was estimated by applying the pooled RR from meta-analysis to the pooled event rate in the control group. As individual participant-level data were not available and follow-up durations varied across trials, precise person-time data were lacking. Therefore, we could not derive robust estimates of incidence rates or event rates per year. Number needed to treat (NNT) was calculated as the reciprocal of the pooled ARR. Heterogeneity was evaluated using the *I^2^* statistic and regarded as high if the value was >50%. An *I^2^* value of 0% would indicate that there were insufficient studies to accurately determine heterogeneity. We performed univariate meta-regression with random effects modelling to study the effect of individual covariates on the outcome. The covariates considered were age, percentage male, body mass index, and length of follow-up. We conducted two subgroup analyses: (i) in patients without baseline HF when it was reported by trials, and (ii) for semaglutide because it was the only GLP-1 receptor agonist investigated more than once in the included RCTs. We conducted a sensitivity analysis excluding the FLOW trial from the primary meta-analysis because it required both type 2 diabetes and chronic kidney disease for eligibility.^11^ Publication bias was not examined by funnel plots and Egger’s test because there were only eleven RCTs included in the largest subgroup, and this was considered too small for a funnel plot to be informative. All analyses were conducted using R software (version 3.6.3).^12^

## Results

We reviewed 1 967 unique records and included 12 RCTs comprising 95 023 patients and 2 901 HF events (Supplementary Figure S1; Supplementary Table S1).^11,13–23^ Eleven RCTs included patients with type 2 diabetes, with 77 419 patients and 2 682 HF events.^11,14–23^ The SELECT trial included 17 604 patients with obesity but without diabetes with 219 HF events.^13^ Six RCTs reported HF events in patients without baseline HF, including 52 753 with 1207 HF events.^11,13–15,21,22^ HF events per study are summarized in Supplementary Table S1. The different GLP-1 receptor agonists investigated are summarized in Supplementary Table S2, while Supplementary Table S3 outlines the definition of HF events and adjudication method.

### Trial characteristics

All trials included a majority of male participants, the median age was between 60 and 66 years, and the median BMI ranged from 30.1 to 33.4 (*Table 1*, Supplementary Table S4). Trials enrolled patients at high cardiovascular risk, including patients with established cardiovascular disease, or multiple cardiovascular risk factors (Supplementary Table S5), and the FLOW trial enrolled patients with type 2 diabetes and chronic kidney disease.^11^

**Table 1:** Trial characteristics

Study name (year)	Intervention	Doses	Indication	Participants (*n*)	Male (%)	Age (years)	BMI (kg/m^2^)	HF (%)	DM (%)	CKD (%)	HTN (%)
**AMPLITUDE-O (2021)**	Efpeglenatide	4 mg or 6 mg/week	Type 2 diabetes	4076	67.0	64.5	32.7	18.0	100	31.7	91.4
**ELIXA (2015)**	Lixisenatide	up to 20μg/day	Type 2 diabetes	6068	66.3	60.3	30.2	22.4	100	*N*/A	76.4
**EXSCEL (2017)**	Exenatide	2 mg/week	Type 2 diabetes	14 752	62.0	62.0	31.7	16.2	100	N/A	N/A
**FLOW (2024)**	Semaglutide	1.0 mg/week	Type 2 diabetes	3533	69.7	66.6	32.0	19.2	100	79.6	N/A
**FREEDOM-CVO (2022)**	Exenatide	Up to 60 mcg/day	Type 2 diabetes	4156	63.3	63.0	32.2	16.1	100	N/A	N/A
**Harmony Outcomes (2018)**	Albiglutide	30–50 mg/week	Type 2 diabetes	9463	69.0	64.1	32.3	20.0	100	N/A	86.5
**LEADER (2016)**	Liraglutide	1.8 mg/day	Type 2 diabetes	9340	64.2	64.3	32.5	17.9	100	24.7	N/A
**PIONEER 6 (2019)**	Semaglutide	up to 14 mg/day	Type 2 diabetes	3183	68.4	66.0	32.3	12.2	100	26.9	N/A
**REWIND (2019)**	Dulaglutide	1.5 mg/week	Type 2 diabetes	9901	53.7	66.2	32.3	8.6	100	22.2	93.1
**SELECT (2023)**	Semaglutide	2.4 mg/week	Obesity	17 604	72.3	61.6	33.3	24.4	0	N/A	81.7
**SOUL (2025)**	Semaglutide	up to 14 mg daily	Type 2 diabetes	9650	71.1	66.1	31.1	23.1	100	42.4	90.8
**SUSTAIN 6 (2016)**	Semaglutide	0.5 mg or 1.0 mg/week	Type 2 diabetes	3297	60.7	64.6	32.8	23.6	100	28.2	92.8

AMPLITUDE-O, Effect of Efpeglenatide on Cardiovascular Outcomes; BMI, Body Mass Index; CKD, Chronic Kidney Disease; CV, Cardiovascular; DM, Diabetes Mellitus; ELIXA, Evaluation of Lixisenatide in Acute Coronary Syndrome; EXSCEL, Exenatide Study of Cardiovascular Event Lowering; FLOW, Evaluate Renal Function with Semaglutide Once Weekly; FREEDOM-CVO, Subcutaneous infusion of exenatide and cardiovascular outcomes in type 2 diabetes; Harmony Outcomes, Albiglutide and cardiovascular outcomes in patients with type 2 diabetes and cardiovascular disease; HF, Heart Failure; HTN, Hypertension; LEADER, Liraglutide Effect and Action in Diabetes: Evaluation of Cardiovascular Outcome Results; PIONEER 6, Peptide Innovation for Early Diabetes Treatment 6; REWIND, Researching Cardiovascular Events with a Weekly Incretin in Diabetes; SELECT, Semaglutide Effects on Cardiovascular Outcomes in People with Overweight or Obesity; SOUL, Semaglutide Cardiovascular Outcomes Trial; SUSTAIN-6, Evaluate Cardiovascular and Other Long-term Outcomes with Semaglutide in Subjects with Type 2 Diabetes; N/A, not available.

Between a sixth and a quarter of patients had baseline HF, with the exception of the REWIND RCT (where fewer than 9% of patients had baseline HF).^14^ When reported, hypertension was highly prevalent amongst participants across all trials, with over 90% of participants reported to have hypertension in the REWIND, AMPLITUDE-O, SUSTAIN 6, and SOUL RCTs (*Table 1*).^14,16,17,19^

Semaglutide was the most frequently studied GLP-1 receptor agonist in the included RCTs (five RCTs, total 37 267 patients and 998 HF events)^11,13,17–19^, with no other GLP-1 receptor agonist tested in more than one of the included RCTs.

The definition of HF events was similar across RCTs, requiring an unscheduled admission to hospital for HF, with the exception of FLOW, SELECT, and SOUL, where an urgent visit to clinic, office, or emergency department with intensification of oral or use of intravenous drugs was included within the endpoint (Supplementary Table S3).^11,13,19^

All RCTs were placebo-controlled, with all events adjudicated by an independent events committee. Risk of bias was low or medium for all RCTs, with only some concerns on measurement of outcome for four RCTs (Supplementary Figures S2 and S3).^11,13,19,22^

### Effect of GLP-1 receptor agonists on heart failure events

**Figure 1:**
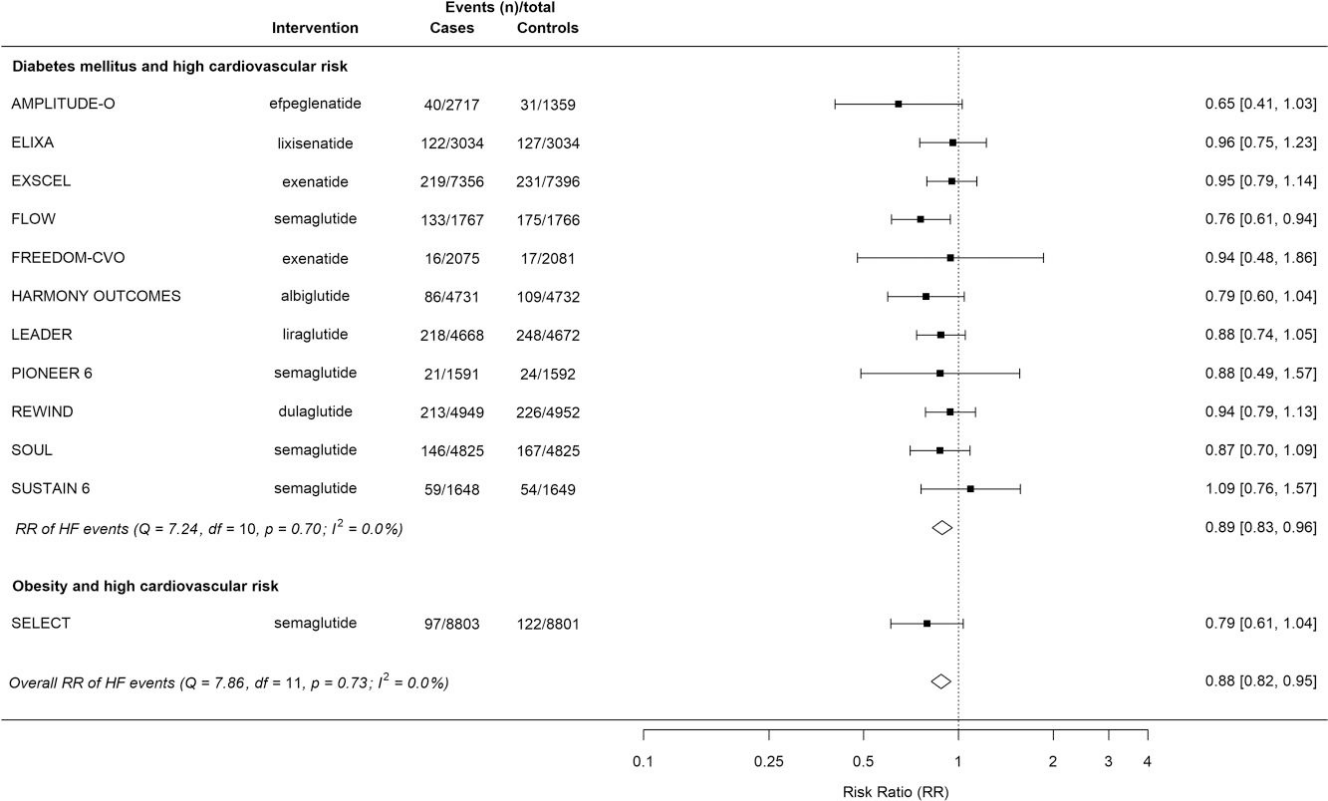
Summary plot of GLP-1 receptor agonists for heart failure events, stratified by indication. AMPLITUDE-O, Effect of Efpeglenatide on Cardiovascular Outcomes; ELIXA, Evaluation of Lixisenatide in Acute Coronary Syndrome; EXSCEL, Exenatide Study of Cardiovascular Event Lowering; FLOW, Evaluate Renal Function with Semaglutide Once Weekly; FREEDOM-CVO, Subcutaneous infusion of exenatide and cardiovascular outcomes in type 2 diabetes; Harmony Outcomes, Albiglutide and cardiovascular outcomes in patients with type 2 diabetes and cardiovascular disease; HF, heart failure; LEADER, Liraglutide Effect and Action in Diabetes: Evaluation of Cardiovascular Outcome Results; PIONEER 6, Peptide Innovation for Early Diabetes Treatment 6; REWIND, Researching Cardiovascular Events with a Weekly Incretin in Diabetes; SELECT, Semaglutide Effects on Cardiovascular Outcomes in People with Overweight or Obesity; SOUL, Semaglutide Cardiovascular Outcomes Trial; SUSTAIN-6, Evaluate Cardiovascular and Other Long-term Outcomes with Semaglutide in Subjects with Type 2 Diabetes

Overall GLP-1 receptor agonist therapy, compared with placebo, reduced the risk of HF events by 12% (RR 0.88, 95% CI 0.82–0.95, ARR 0.42%, 95% CI 0.17%-0.62%; NNT 238, 95% CI 161–588) (*Figure 1*).

### Effect of GLP-1 receptor agonists on heart failure events in patients with diabetes

In patients with diabetes, GLP-1 receptor agonist therapy compared with placebo reduced the risk of HF events by 11% (RR 0.89, 95% CI 0.83–0.96; ARR 0.40%, 95% CI 0.15–0.62; NNT 250, 95% CI 161–667) (*Figure 1*).

### Effect of GLP-1 receptor agonists on heart failure events in patients with obesity without diabetes

In the SELECT RCT, the risk of HF events was numerically reduced, but did not reach statistical significance (RR 0.79, 95% CI 0.61–1.04) (*Figure 1*), with the trial powered for a primary outcome of major adverse cardiovascular events (death from cardiovascular causes, nonfatal myocardial infarction, or nonfatal stroke).^13^

### Subgroup analysis

#### Effect of GLP-1 receptor agonists in patients without baseline heart failure

**Figure 2:**
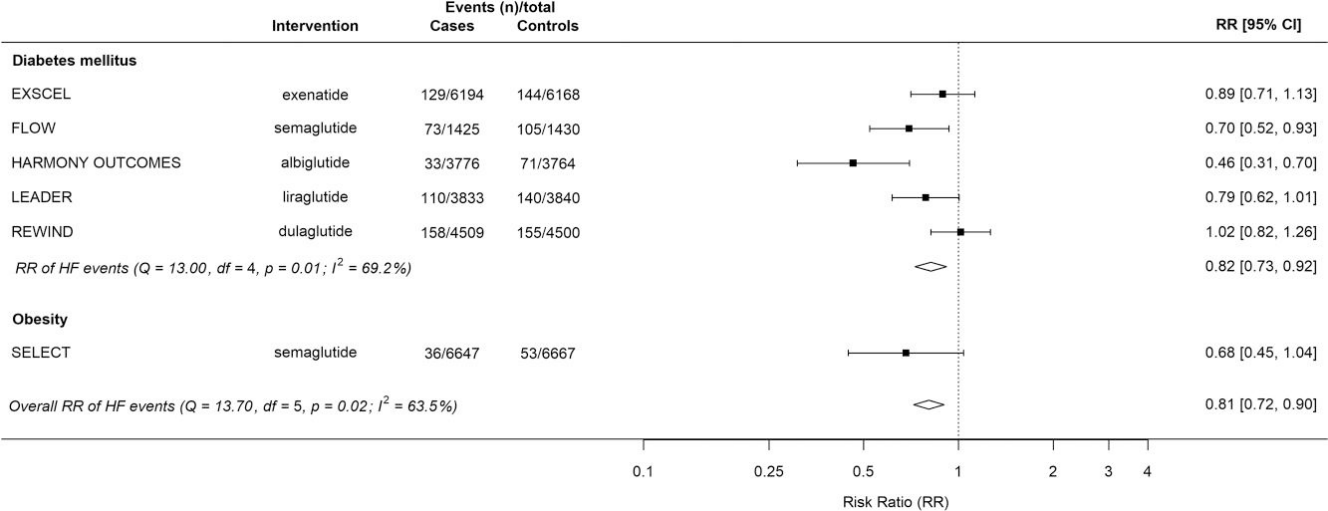
Summary plot for GLP-1 receptor agonists for heart failure events in patients without baseline heart failure, stratified by indication. EXSCEL, Exenatide Study of Cardiovascular Event Lowering; FLOW, Evaluate Renal Function with Semaglutide Once Weekly; Harmony Outcomes, Albiglutide and cardiovascular outcomes in patients with type 2 diabetes and cardiovascular disease; HF, heart failure; LEADER, Liraglutide Effect and Action in Diabetes: Evaluation of Cardiovascular Outcome Results; REWIND, Researching Cardiovascular Events with a Weekly Incretin in Diabetes; SELECT, Semaglutide Effects on Cardiovascular Outcomes in People with Overweight or Obesity

In patients without baseline HF, HF events were reduced by 19% by GLP-1 receptor agonist therapy compared with placebo (RR 0.81, 95% CI 0.72–0.90; ARR 0.60%, 95% CI 0.32%-0.89%; NNT 167, 95% CI 113–313) (*Figure 2*).

#### Effect of GLP-1 receptor agonists in patients with diabetes without baseline heart failure

In patients with diabetes but without baseline HF, GLP-1 receptor agonist therapy reduced HF events by 18% compared with placebo (RR 0.82, 95% CI 0.73–0.92; ARR 0.66%, 95% CI 0.29%–0.99%; NNT 152, 95% CI 101–345) (*Figure 2*).

#### Effect of GLP-1 receptor agonists in patients with obesity without diabetes and without baseline heart failure

In the SELECT RCT, with 13 314 patients without baseline HF and 89 HF events, the risk of HF events was numerically reduced by 32%, but this did not reach statistical significance (RR 0.68, 95% CI 0.45–1.04) (*Figure 2*).^13^

#### Effect of semaglutide on heart failure events

**Figure 3:**
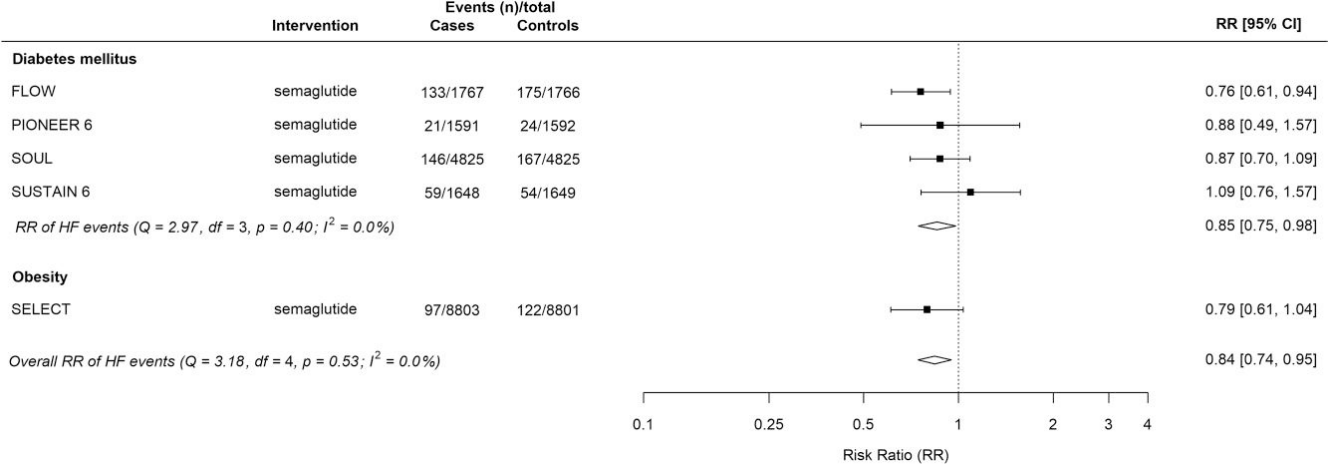
Summary plot of semaglutide for heart failure events, stratified by indication. FLOW, Evaluate Renal Function with Semaglutide Once Weekly; HF, heart failure; PIONEER 6, Peptide Innovation for Early Diabetes Treatment 6; SELECT, Semaglutide Effects on Cardiovascular Outcomes in People with Overweight or Obesity; SOUL, Semaglutide Cardiovascular Outcomes Trial; SUSTAIN-6, Evaluate Cardiovascular and Other Long-term Outcomes with Semaglutide in Subjects with Type 2 Diabetes

Semaglutide reduced the risk of HF events by 16% compared with placebo (RR 0.84, 95% CI 0.74–0.95; ARR 0.62%, 95% CI 0.19%-1.00%; NNT 161, 95% CI 100–526) (*Figure 3*). In patients without baseline HF across the FLOW and SELECT RCTs (16 169 patients, 267 events) semaglutide reduced the risk of HF events by 31% (RR 0.69, 95% CI 0.55–0.88; ARR 1.25%, 95% CI 0.48%-1.82%; NNT 80, 95% CI 55–208) (Supplementary Figure S4).^11,13^

### Meta-regression

Meta-regression demonstrated no variation in the effect of GLP-1 receptor agonist therapy for HF events by age, body mass index, or duration of follow-up (Supplementary Table S6). There was no difference in the effect of semaglutide on HF events compared with other GLP-1 receptor agonists (RR 0.93, 0.81–1.07).

### Sensitivity analysis

When excluding the FLOW trial, the reduction observed in HF events with GLP-1 receptor agonist therapy remained (Supplementary Figure S5).

### Safety outcomes

In pooled analyses, the safety outcomes of risk of acute pancreatitis, severe hypoglycaemia, acute renal failure and cancer was not higher in patients randomized to receive GLP-1 receptor agonists, including semaglutide specifically, than those randomized to receive placebo (Supplementary Table S7, Supplementary Figures S6–S13).

## Discussion

This contemporary meta-analysis of data from twelve cardiovascular outcomes RCTs, including over 95 000 patients affirms that GLP-1 receptor agonist therapy reduces risk of HF events in patients with diabetes and/or obesity by 12%, but finds that the absolute risk reduction across the population was only 0.42% (NNT 238). When limiting to those without baseline HF, a greater relative risk (19%) and absolute risk (0.60%) reduction was observed (NNT 167). Semaglutide was especially efficacious, conferring a 1.25% absolute risk reduction of HF events in patients without baseline HF (NNT 80).

### Comparison with previous individual studies

Our results extend beyond previous meta-analyses which summarized HF events with GLP-1 receptor agonist therapy in patients with diabetes and/or obesity.^24–29^ Firstly, in this meta-analysis we resolve previously conflicting results by verifying that GLP-1 receptor agonist moderately reduces relative risk of HF events in this population. Second, we provide estimates for the absolute reduction in risk of HF events observed with GLP-1 receptor agonist therapy, including for baseline HF, which is a necessary clinical nuance for patients and healthcare providers. Third, we demonstrate that reduction of HF events is also seen specifically for treatment with semaglutide. This finding is especially important because evidence for other agents is either conflicting (i.e. tirzepatide) or lacking.^25,30^

### Comparison of GLP-1 receptor agonists with mainstay medicines for heart failure prevention

Our NNT estimates must be contextualized with the first-line preventive medicines for HF events in those with obesity and/or diabetes. For diabetes, sodium-glucose cotransporter-2 inhibitors (SGLT2is) appear to offer greater absolute preventive effect. In those with diabetes, the DECLARE-TIMI-58 trial (*N* = 17 160 patients) demonstrated dapagliflozin had an NNT of 43 for preventing HF hospitalization.^31^ Similarly, in the smaller EMPA-REG OUTCOME trial (*N* = 7 020 patients), empagliflozin had an NNT of 71 to prevent HF hospitalization.^32^ The totality of evidence suggests that in those with diabetes, SGLT2 inhibitors offer greater tangible clinical benefit with respect to HF event prevention.

### GLP-1 receptor agonists and progression to symptomatic heart failure

GLP-1 receptor agonists may reduce the development of HF by direct and indirect effects on the progression from pre- to symptomatic HF (stage B to C). In terms of direct effects, GLP-1 receptor agonists reduce inflammation, oxidative stress, and renin-angiotensin-aldosterone upregulation.^33,34^ Furthermore, GLP-1 receptor agonists reduce ischaemic events, which can lead to deterioration in left ventricular systolic function.^9,35^ Notably, we did not find a variation in the reduction of HF events by median body mass index, and in the HARMONY outcomes trial, there was only a small impact on glycaemic control and weight loss relative to the impact on HF events.^22^ In addition, weight loss in patients with obesity but no HF is associated with reversal of hypertrophic chamber remodelling, improvement in ventricular mechanics, and reductions in haemodynamic congestion.^36–38^ In patients with obesity-related HF with preserved ejection fraction (HFpEF) semaglutide has been shown to reduce natriuretic peptide levels, left atrial volumes, right ventricular dimensions, and transmitral E-wave velocity,^39^ highlighting a potential decongestive effect that may also delay manifestation of the HF syndrome.^40^ Previous analyses have demonstrated that reduction in HF events with GLP-1 receptor agonists in patients with obesity-related HFpEF was greater with higher initial body mass index and a greater magnitude of weight loss.^41^ Notably, there are conflicting results for the effects of GLP-1 receptor agonists in patients with HFrEF,^42^ though our analysis could not distinguish between presentations of HFpEF and HFrEF.^1^

### Implications for clinicians, policy makers, and future research

The prevalence of diabetes and obesity has reached epidemic proportions.^43,44^ Among patients with preclinical HF, uncontrolled diabetes is associated with a substantial risk of HF progression.^45^ Uncorrected obesity has been shown to provoke pathological alteration in myocardium encompassing cardiomegaly, tissue fibrosis, deranged force generation and relaxation, in conjunction with endothelial dysfunction, which all predispose to deterioration to symptomatic HF. Compared to other non-communicable diseases, the cost of HF care is estimated to significantly increase, the majority of which is related to unplanned care.^2^ Thus, the burden of diabetes and obesity-related HF on health systems will grow, suggesting that targeting diabetes and obesity early in the HF continuum is critical.

GLP-1 receptor agonists are already recommended by the European Society of Cardiology guidelines for their cardiovascular risk reduction in patients with type 2 diabetes and established cardiovascular or kidney disease, or multiple risk factors for cardiovascular disease.^46,47^ Our analyses provide evidence that GLP-1 receptor agonists reduce the progression from pre-clinical to clinical HF, albeit with small absolute risk reduction due to a low baseline event rate. Notably, a pre-specified analysis of the FLOW trial showed that, in patients with type 2 diabetes and chronic kidney disease, the number needed to treat to prevent one HF event was 49 over 3 years,^48^ suggesting that for these patients with multiple morbidities associated with HF genesis, the absolute benefits may be more substantial.

### Limitations

Our study results must be viewed within the context of several limitations. First, we studied HF events as an outcome that is important to health systems and patients, but we do not have data reported from the trials for HF diagnoses that were made outside of the hospital setting or were not the primary reason for hospital attendance. It is possible that the magnitude of protective effect seen for GLP-1 receptor agonists may vary by mode of HF presentation. Second, we do not have data for whether the HF events were attributed to presentations for HFrEF or HFpEF. Third, for the analysis restricted to patients without baseline HF, only six of the twelve trial reported HF status stratified analyses, which offers a smaller sample size. Fourth, this is a study-level meta-analysis, and no patient-level data were available. Fifth, while we evaluated baseline BMI, age, and follow-up duration with meta-regression, we did not evaluate whether the effect of GLP-1 benefit is independent of weight change during treatment. Sixth, only one trial (SELECT) included patients with obesity without co-existent diabetes.^13^ Thus, we do not have adequate data to determine if GLP-1 receptor agonists can prevent HF events in patients with obesity but without diabetes. The SURMOUNT-MMO trial (NCT05556512), enrolling ∼15 000 participants with obesity without diabetes for the five-component composite of nonfatal myocardial infarction, nonfatal stroke, coronary revascularization, HF events, or death from any cause, will help address this evidence gap when it reports in 2027.^49^

## Conclusion

GLP-1 receptor agonists have a moderate relative benefit but a small absolute benefit for reduction of heart failure events in patients with type 2 diabetes and/or obesity, including in those without baseline heart failure.

## Supplementary Material

xvag091_Supplementary_Data

## Declarations

### Data Availability

Data are available on reasonable request. Technical appendix, statistical code and dataset are available from the corresponding author at r.nadarajah@leeds.ac.uk

### Pre-registered Clinical Trial Number

None supplied.

### Personal Thanks

JW was supported by Barts Charity (MGU0504).

## Prior presentation

This work has been presented at the European Society of Cardiology Congress 2025, Madrid, Spain.
