# Supplementary material for: Glucagon-like peptide-1 receptor agonists for prevention of heart failure events in type 2 diabetes and/or obesity
Source: ESC Heart Fail. 2026 Mar 25;13(2):xvag091. doi: 10.1093/eschf/xvag091 (PMC13064920; doi:10.1093/eschf/xvag091)
Supplement: xvag091_Supplementary_Data [file xvag091_supplementary_data.docx]

**Supplementary Appendix**

Glucagon-like peptide-1 receptor agonists for prevention of heart failure events in patients with type 2 diabetes and/or obesity: a systematic review and meta-analysis of randomised controlled trials

# **Contents**

| Section 1: Medline Search Strategy | 3 |
| --- | --- |
| Section 2: OVID EMBASE Search Strategy | 4 |
| Section 3: Cochrane Library Search Strategy | 6 |
| Section 4: Definitions | 7 |
| Supplementary Table 1: Heart Failure Events per study | 8 |
| Supplementary Table 2: Drugs, preparations and outcomes | 9 |
| Supplementary Table 3: Definition of HF events per trial and adjudication method | 11 |
| Supplementary Table 4: Participant Baseline Characteristics | 12 |
| Supplementary Table 5: Extended Study Characteristics and Endpoint definitions | 13 |
| Supplementary Table 6: Meta-regression | 15 |
| Supplementary Table 7: Adverse Events per study | 16 |
| Supplementary Figure 1: The PRISMA flowchart of studies included for systematic review and meta-analysis | 17 |
| Supplementary Figure 2: Risk of Bias per study | 18 |
| Supplementary Figure 3: Summary plot of Risk of Bias | 19 |
| Supplementary Figure 4: Summary plot of GLP-1 receptor agonists in patients without baseline heart failure across the FLOW and SELECT trials, stratified by indication | 20 |
| Supplementary Figure 5: Summary plot of GLP-1 receptor agonists for heart failure events excluding the FLOW trial, stratified by indication | 21 |
| Supplementary Figure 6: Summary plot of GLP-1 receptor agonist safety outcomes; acute pancreatitis | 22 |
| Supplementary Figure 7: Summary plot of GLP-1 receptor agonist safety outcomes; severe hypoglycaemia | 23 |
| Supplementary Figure 8: Summary plot of GLP-1 receptor agonist safety outcomes; acute renal failure | 24 |
| Supplementary Figure 9: Summary plot of GLP-1 receptor agonist safety outcomes; cancer | 25 |
| Supplementary Figure 10: Summary plot of Semaglutide specific safety outcomes; acute pancreatitis | 26 |
| Supplementary Figure 11: Summary plot of Semaglutide specific safety outcomes; severe hypoglycaemia | 27 |
| Supplementary Figure 12: Summary plot of Semaglutide specific safety outcomes; acute renal failure | 28 |
| Supplementary Figure 13: Summary plot of Semaglutide specific safety outcomes; cancer | 29 |
| PRISMA Abstract Checklist | 30 |
| PRISMA Manuscript Chekclist | 31 |

# **Section 1: Medline Search Strategy**

|  | Medline |  |
| --- | --- | --- |
| 1 | Heart failure/ | 156177 |
| 2 | ((heart or cardiac or congestive) adj7 fail*).ti,ab. | 259081 |
| 3 | Hf.ti,ab. | 71058 |
| 4 | 1-3 | 329853 |
| 5 | exenatide/ or liraglutide/ or albiglutide/ or dulaglutide/ or semaglutide/ or lixisenatide/ or tirzepatide/ | 5886 |
| 5 | (glucagon like peptide 1 receptor agonist or GLP* or exenatide or liraglutide or lixisenatide or dulaglutide or semaglutide or retatrutide or orforglipron or danuglipron).tw. | 29641 |
| 7 | 5 or 6 | 30370 |
| 8 | exp randomized controlled trial/ | 639593 |
| 9 | controlled clinical trial.pt. | 95685 |
| 10 | randomi?ed.ab. | 823925 |
| 11 | placebo.ab. | 258360 |
| 12 | drug therapy.fs. | 2812234 |
| 13 | randomly.ab. | 459521 |
| 14 | trial.ab. | 752477 |
| 15 | groups.ab. | 2849830 |
| 16 | or/8-15 | 6309457 |
| 17 | 4 or 7 or 16 | 983 |
| 18 | exp animals/ not humans.sh. | 5338433 |
| 19 | 17 not 18 | 898 |
| 20 | 19 not ((exp infant/ or exp child/ or adolescent/) not exp adult/) | 897 |

# **Section 2: OVID EMBASE Search**

|  | EMBASE |  |
| --- | --- | --- |
| 1 | heart failure/ | 370865 |
| 2 | ((heart or cardiac or congestive) adj7 fail*).ti,ab. | 448868 |
| 3 | hf.ti,ab. | 122099 |
| 4 | or/1-3 | 603299 |
| 5 | exenatide/ or liraglutide/ or albiglutide/ or dulaglutide/ or semaglutide/ or lixisenatide/ or tirzepatide/ | 30010 |
| 6 | (glucagon like peptide 1 receptor agonist or GLP* or exenatide or liraglutide or lixisenatide or dulaglutide or semaglutide or retatrutide or orforglipron or danuglipron).tw. | 49467 |
| 7 | 5 or 6 | 59028 |
| 8 | exp randomized controlled trial/ | 884512 |
| 9 | Controlled clinical trial/ | 445804 |
| 10 | random$.ti,ab. | 2212984 |
| 11 | randomization/ | 100902 |
| 12 | intermethod comparison/ | 313928 |
| 13 | placebo.ti,ab. | 397374 |
| 14 | (compare or compared or comparison).ti. | 687364 |
| 15 | ((evaluated or evaluate or evaluating or assessed or assess) and (compare or compared or comparing or comparison)).ab. | 3135882 |
| 16 | (open adj label).ti,ab. | 123512 |
| 17 | ((double or single or doubly or singly) adj (blind or blinded or blindly)).ti,ab. | 299724 |
| 18 | double blind procedure/ | 234030 |
| 19 | parallel group$1.ti,ab. | 35413 |
| 20 | (crossover or cross over).ti,ab. | 135087 |
| 21 | ((assign$ or match or matched or allocation) adj5 (alternate or group$1 or intervention$1 or patient$1 or subject$1 or participant$1)).ti,ab. | 460346 |
| 22 | (assigned or allocated).ti,ab. | 544619 |
| 23 | (controlled adj7 (study or design or trial)).ti,ab. | 506521 |
| 24 | (volunteer or volunteers).ti,ab. | 304317 |
| 25 | human experiment/ | 690712 |
| 26 | trial.ti. | 466416 |
| 27 | or/8-26 | 7054410 |
| 28 | (random$ adj sampl$ adj7 ("cross section$" or questionnaire$1 or survey$ or database$1)).ti,ab. not (comparative study/ or controlled study/ or randomi?ed controlled.ti,ab. or randomly assigned.ti,ab.) | 10529 |
| 29 | Cross-sectional study/ not (exp randomized controlled trial/ or controlled clinical study/ or controlled study/ or randomi?ed controlled.ti,ab. or control group$1.ti,ab.) | 439635 |
| 30 | (((case adj control$) and random$) not randomi?ed controlled).ti,ab. | 23504 |
| 31 | Systematic review.ti,ab. not (trial or study).ti. | 399750 |
| 32 | (nonrandom$ not random$).ti,ab. | 20290 |
| 33 | "random field$".ti,ab. | 3154 |
| 34 | (random cluster adj3 sampl$).ti,ab. | 1745 |
| 35 | (review.ab. and review.pt.) not trial.ti. | 1280778 |
| 36 | "we searched".ab. and (review.ti. or review.pt.) | 57764 |
| 37 | "update review".ab. | 153 |
| 38 | (databases adj4 searched).ab. | 75860 |
| 39 | (rat or rats or mouse or mice or swine or porcine or murine or sheep or lambs or pigs or piglets or rabbit or rabbits or cat or cats or dog or dogs or cattle or bovine or monkey or monkeys or trout or marmoset$1).ti. and animal experiment/ | 1298534 |
| 40 | Animal experiment/ not (human experiment/ or human/) | 2741074 |
| 41 | or/28-40 | 4853468 |
| 42 | 27 not 41 | 6188563 |
| 43 | 4 and 7 and 42 | 1035 |

# **Section 3: Cochrane Library Search Strategy**

|  | Cochrane |  |
| --- | --- | --- |
| 1 | [mh "heart failure"] | 14786 |
| 2 | HF:ti,ab | 12360 |
| 3 | ((heart or cardiac or congestive) NEAR/7 fail*):ti,ab | 37153 |
| 4 | #1 or #2 or #3 | 42269 |
| 5 | [mh "Glucagon-Like Peptide-1 Receptor Agonists"] | 296 |
| 6 | (glucagon like peptide 1 receptor agonist or GLP* or exenatide or liraglutide or lixisenatide or dulaglutide or semaglutide or retatrutide or orforglipron or danuglipron):ti,ab | 9671 |
| 7 | 5 or 6 | 9678 |
| 8 | 4 and 7 | 347 |

# **Section 4: Definition**

**Study designs** - We included randomised clinical trials reporting heart failure events in patients randomised to a GLP-1 receptor agonist compared to placebo

**PICO**

| Randomised clinical trials with an intervention and control arm |  |
| --- | --- |
| Patient, problem, or population | People aged ≥18 years |
| Intervention | GLP-1 receptor agonist |
| Comparison, control, or comparator | Placebo |
| Outcome | Heart failure events |

**Risk of Bias assessment**

We used the RoB2 tool produce by Cochrane Reviews to assess risk of bias in the included studies. The RoB2 tool assesses risk in the following categories:

1. Bias arising from the randomization process
2. Bias due to deviations from intended interventions
3. Bias due to missing outcome data
4. Bias in measurement of the outcome
5. Bias in selection of the reported result

The tool provides ‘signalling questions’ which the researcher answers allowing the tool to formulate a judgement regarding the level of bias for each category. Potential answers include:

- Yes
- Probably yes
- Probably no
- No
- No information

The tool then produces a risk of bias judgement:

- Low risk of bias
- Some concerns
- High risk of bias

The trials are then assigned an overall score based on the following criteria:

- Low risk: If all domains scored ‘low risk’ of bias
- Moderate risk: If < 3 domains scored ‘some concerns’
- High risk: If 1 or more domain scored ‘high risk’ of bias.

# **Supplementary Table 1: Heart Failure Events per study**

| **Study Name** | **Control arm (n)** | **Intervention arm (n)** |
| --- | --- | --- |
| **AMPLITUDE-O** | 31 | 40 |
| **ELIXA** | 127 | 122 |
| **EXSCEL** | 231 | 219 |
| **FLOW** | 175 | 133 |
| **FREEDOM-CVO** | 17 | 16 |
| **Harmony Outcomes** | 109 | 86 |
| **LEADER** | 248 | 218 |
| **PIONEER 6** | 24 | 21 |
| **SELECT** | 122 | 97 |
| **REWIND** | 226 | 213 |
| **SOUL** | 167 | 146 |
| **SUSTAIN 6** | 54 | 59 |

AMPLITUDE-O - Effect of Efpeglenatide on Cardiovascular Outcomes; ELIXA - Evaluation of Lixisenatide in Acute Coronary Syndrome; EXSCEL - Exenatide Study of Cardiovascular Event Lowering; FLOW - Evaluate Renal Function with Semaglutide Once Weekly; FREEDOM-CVO - Subcutaneous infusion of exenatide and cardiovascular outcomes in type 2 diabetes; Harmony Outcomes - Albiglutide and cardiovascular outcomes in patients with type 2 diabetes and cardiovascular disease; LEADER - Liraglutide Effect and Action in Diabetes: Evaluation of Cardiovascular Outcome Results; PIONEER 6 - Peptide Innovation for Early Diabetes Treatment 6; REWIND - Researching Cardiovascular Events with a Weekly Incretin in Diabetes; SELECT - Semaglutide Effects on Cardiovascular Outcomes in People with Overweight or Obesity; SOUL - Semaglutide Cardiovascular Outcomes Trial; SUSTAIN-6 - Evaluate Cardiovascular and Other Long-term Outcomes with Semaglutide in Subjects with Type 2 Diabetes.

# **Supplementary Table 2: Drugs, preparations and outcomes**

| **Study Name** | **Intervention** | **Preparation** | **Doses** | **Primary Outcome** | **Secondary Outcome(s)** |
| --- | --- | --- | --- | --- | --- |
| **AMPLITUDE-O** | Efpeglenatide | SC | 4mg or 6mg / week | Composite of nonfatal MI, nonfatal stroke or CV death | Composite of MACE, coronary revascularization or hospitalization for unstable angina; composite of new macroalbuminuria + increased UACR of ≥30% from baseline, eGFR decrease by ≥ 40% for ≥30 days, or end stage renal disease |
| **ELIXA** | Lixisenatide | SC | up to 20 μg / day | Composite of CV death, nonfatal MI, nonfatal stroke, hospitalization for unstable angina | Composite of primary outcome or hospitalization for HF, composite of primary outcome or hospitalization for HF or hospitalization for coronary revascularization procedure |
| **EXSCEL** | Exenatide | SC | 2mg / week | Composite of nonfatal myocardial infarction, nonfatal stroke or CV death | All-cause mortality, non-fatal MI, non-datal stroke, CV death, hospitalization for ACS, hospitalization for HF |
| **FLOW** | Semaglutide | SC | 1.0mg / week | Composite of onset of kidney failure, sustained 50% or greater reduction in eGFR from baseline, or death from kidney-related or CV causes | Total eGFR slope (the annual rate of change in eGFR from randomization to the end of the trial), MACE, all-cause death |
| **FREEDOM-CVO** | Exenatide | SC | 60micrograms / day | Composite of CV death, nonfatal MI, nonfatal stroke or hospitalization for unstable angina | All-cause death, CV death, nonfatal MI, nonfatal stroke, hospitalization for unstable angina, hospitalization for HF |
| **Harmony Outcomes** | Albiglutide | SC | 30-50mg / week | Composite of CV death, nonfatal MI and nonfatal stroke | Composite of the primary outcome with urgent revascularization for unstable angina, CV death, MI, stroke, composite of CV death and hospitalization for HF |
| **LEADER** | Liraglutide | SC | 1.8mg / day | Composite of CV death, nonfatal MI or nonfatal stroke | Composite of CV death, nonfatal MI, nonfatal stroke, coronary revascularization or hospitalization for unstable angina or HF; all-cause mortality; composite renal and retinal microvascular outcome (nephropathy and retinopathy); neoplasms and pancreatitis |
| **PIONEER 6** | Semaglutide | oral | up to 14mg / day | Composite of CV death, nonfatal MI or nonfatal stroke | Composite of the primary outcome plus unstable angina resulting in hospitalization or HF resulting in hospitalization; composite of all-cause death, nonfatal MI nonfatal stroke; individual components of the composite outcomes |
| **REWIND** | Dulaglutide | SC | 1.5mg / week | Composite of CV death or death from unknown causes, nonfatal MI or nonfatal stroke | Composite of diabetic retinopathy or renal disease; hospital admission for unstable angina; each component of the primary composite CV outcome; death; and HF requiring either hospital admission or an urgent visit requiring therapy |
| **SELECT** | Semaglutide | SC | 2.4mg / week | Composite of death CV causes, nonfatal MI or nonfatal stroke | CV death; a composite of CV death or HF requiring hospitalization or urgent medical visit; all-cause death |
| **SOUL** | Semaglutide | oral | up to 14mg daily | Composite of death CV causes, nonfatal MI or nonfatal stroke | Composite of CV death, renal death, onset of persistent 50% or more reduction in eGFR; CV death; HF requiring hospitalization; urgent HF visit |
| **SUSTAIN 6** | Semaglutide | SC | 0.5mg or 1.0mg / week | Composite of death CV causes, nonfatal MI or nonfatal stroke | Composite of CV death, nonfatal MI, nonfatal stroke, coronary or peripheral revascularization, and hospitalization for unstable angina or HF; composite of all-cause death, nonfatal MI or nonfatal stroke; the individual components of the composite outcomes |

ACS - acute coronary syndrome; BMI – Body Mass Index; CKD – chronic kidney disease; CV – cardiovascular; DM – Diabetes Mellitus; eGFR – estimated Glomerular Filtration Rate; ELIXA - Evaluation of Lixisenatide in Acute Coronary Syndrome; EXSCEL - Exenatide Study of Cardiovascular Event Lowering; FLOW - Evaluate Renal Function with Semaglutide Once Weekly; FREEDOM-CVO - Subcutaneous infusion of exenatide and cardiovascular outcomes in type 2 diabetes; HF – Heart Failure; IHD – ischaemic heart disease; LEADER - Liraglutide Effect and Action in Diabetes: Evaluation of Cardiovascular Outcome Results; MACE – major adverse cardiovascular event; MI – myocardial infarction; PIONEER 6 - Peptide Innovation for Early Diabetes Treatment 6; REWIND - Researching Cardiovascular Events with a Weekly Incretin in Diabetes; SC – subcutaneous; SELECT - Semaglutide Effects on Cardiovascular Outcomes in People with Overweight or Obesity; SOUL - Semaglutide Cardiovascular Outcomes Trial; SUSTAIN-6 - Evaluate Cardiovascular and Other Long-term Outcomes with Semaglutide in Subjects with Type 2 Diabetes; T2D – Type 2 Diabetes Mellitus.

# **Supplementary Table 3: Definition of HF events per trial and adjudication method**

| **Study**  **Name** | **Endpoint definition** | **Event Ascertainment** |
| --- | --- | --- |
| **AMPLITUDE-O** | Hospitalization with a primary diagnosis of HF, length of stay >24 hours, new or worsening symptoms due to HF, objective evidence of new or worsening HF and the patient receives initiation or intensification of treatment specifically for HF | Adjudicated in a blinded fashion by an event adjudication committee |
| **ELIXA** | Unplanned presentation to an acute care facility for a worsening of symptoms and signs of HF requiring an overnight stay | Adjudicated in a blinded fashion by an event adjudication committee |
| **EXSCEL** | Hospitalization with a primary diagnosis of HF, length of stay ≥ 24 hours, new or worsening symptoms due to HF, objective evidence of new or worsening HF, initiation or intensification of HF treatment | Adjudicated in a blinded fashion by an event adjudication committee |
| **FLOW** | New onset or worsening of HF leading to an unscheduled hospital admission or an urgent clinic/office/emergency department visit | Adjudicated in a blinded fashion by an event adjudication committee |
| **FREEDOM-CVO** | Hospitalization with a primary diagnosis of HF, length of stay >24 hours, new or worsening symptoms due to HF and objective evidence of new or worsening HF and initiation or intensification of treatment specifically for HF | Adjudicated in a blinded fashion by an event adjudication committee |
| **Harmony Outcomes** | Hospitalization with a primary diagnosis of HF, length of stay >24 hours, new or worsening symptoms due to HF, objective evidence of new or worsening HF and the patient receives initiation or intensification of treatment specifically for HF | Adjudicated in a blinded fashion by an event adjudication committee |
| **LEADER** | Hospitalization or emergency department visit with a primary diagnosis of heart failure, length of stay ≥12 hours, and at least one clinical manifestation or radiological evidence of worsening HF, initiation or intensification of treatment specifically for HF | Adjudicated in a blinded fashion by an event adjudication committee |
| **PIONEER 6** | Hospitalization with a primary diagnosis of HF, either a new episode or worsening of existing HF | Adjudicated in a blinded fashion by an event adjudication committee |
| **REWIND** | HF requiring hospitalization or an urgent visit requiring initiation or intensification of therapy | Adjudicated in a blinded fashion by an event adjudication committee |
| **SELECT** | Hospitalization or urgent medical visit for HF | Adjudicated in a blinded fashion by an event adjudication committee |
| **SOUL** | Hospitalization with a primary diagnosis of HF, either a new episode or worsening of existing HF | Adjudicated in a blinded fashion by an event adjudication committee |
| **SUSTAIN 6** | Hospitalization with a primary diagnosis of HF, length of stay >24 hours, new or worsening symptoms due to HF, objective evidence of new or worsening HF and the patient receives initiation or intensification of treatment specifically for HF | Adjudicated in a blinded fashion by an event adjudication committee |

AMPLITUDE-O - Effect of Efpeglenatide on Cardiovascular Outcomes; ELIXA - Evaluation of Lixisenatide in Acute Coronary Syndrome; EXSCEL - Exenatide Study of Cardiovascular Event Lowering; FLOW - Evaluate Renal Function with Semaglutide Once Weekly; FREEDOM-CVO - Subcutaneous infusion of exenatide and cardiovascular outcomes in type 2 diabetes; Harmony Outcomes - Albiglutide and cardiovascular outcomes in patients with type 2 diabetes and cardiovascular disease; HF – Heart Failure; LEADER - Liraglutide Effect and Action in Diabetes: Evaluation of Cardiovascular Outcome Results; PIONEER 6 - Peptide Innovation for Early Diabetes Treatment 6; REWIND - Researching Cardiovascular Events with a Weekly Incretin in Diabetes; SELECT - Semaglutide Effects on Cardiovascular Outcomes in People with Overweight or Obesity; SOUL - Semaglutide Cardiovascular Outcomes Trial; SUSTAIN-6 - Evaluate Cardiovascular and Other Long-term Outcomes with Semaglutide in Subjects with Type 2 Diabetes

# **Supplementary Table 4: Participant Baseline Characteristics**

| **Study Name** | **Control (n)** | **Intervention (n)** | **BMI Intervention arm** | **BMI Control arm** | **T2D Intervention arm (%)** | **T2D Control arm (%)** | **HF Intervention arm (%)** | **HF Control arm (%)** | **IHD Intervention arm (%)** | **IHD control arm (%)** | **CKD Intervention arm (%)** | **CKD Control arm (%)** |
| --- | --- | --- | --- | --- | --- | --- | --- | --- | --- | --- | --- | --- |
| **AMPLITUDE-O** | 1359 | 2718 | 32.7 | 32.9 | 100 | 100 | 18.1 | 17.9 | N/A | N/A | 31.6 | 31.8 |
| **ELIXA** | 3034 | 3034 | 30.1 | 30.2 | 100 | 100 | 22.5 | 22.3 | 100 | 100 | N/A | N/A |
| **EXSCEL** | 7396 | 7356 | 31.8 | 31.7 | 100 | 100 | 15.8 | 16.6 | 53.0 | 52.7 | N/A | N/A |
| **FLOW** | 1766 | 1767 | 31.9 | 32.0 | 100 | 100 | 19.4 | 19.0 | N/A | N/A | 79.1 | 80.0 |
| **FREEDOM-CVO** | 2081 | 2075 | 32.4 | 31.9 | 100 | 100 | 15.7 | 16.5 | N/A | N/A | N/A | N/A |
| **Harmony Outcomes** | 4732 | 4731 | 32.3 | 32.3 | 100 | 100 | 20.0 | 20.0 | 70.0 | 71.0 | N/A | N/A |
| **LEADER** | 4672 | 4668 | 32.5 | 32.5 | 100 | 100 | 17.9 | 17.8 | 35.4 | 35.0 | 25.4 | 24.0 |
| **PIONEER 6** | 1592 | 1591 | 32.3 | 32.3 | 100 | 100 | 11.8 | 12.6 | 22.4 | 23.6 | 27.3 | 26.5 |
| **REWIND** | 4952 | 4949 | 32.3 | 32.3 | 100 | 100 | 8.5 | 8.7 | N/A | N/A | 21.8 | 22.6 |
| **SELECT** | 8801 | 8803 | 33.3 | 33.4 | 0 | 0 | 24.5 | 24.2 | 82.2 | 82.0 | N/A | N/A |
| **SOUL** | 4825 | 4825 | 31.0 | 31.2 | 100 | 100 | 22.9 | 23.3 | 70.6 | 70.8 | 42.3 | 42.5 |
| **SUSTAIN 6** | 1649 | 1648 | 32.8 | 32.8 | 100 | 100 | 23.1 | 24.0 | 60.0 | 61.0 | 28.2 | 28.1 |

AMPLITUDE-O - Effect of Efpeglenatide on Cardiovascular Outcomes; BMI – Body Mass Index; CKD – chronic kidney disease; ELIXA - Evaluation of Lixisenatide in Acute Coronary Syndrome; EXSCEL - Exenatide Study of Cardiovascular Event Lowering; FLOW - Evaluate Renal Function with Semaglutide Once Weekly; FREEDOM-CVO - Subcutaneous infusion of exenatide and cardiovascular outcomes in type 2 diabetes; Harmony Outcomes - Albiglutide and cardiovascular outcomes in patients with type 2 diabetes and cardiovascular disease; HF – Heart Failure; IHD – ischaemic heart disease; LEADER - Liraglutide Effect and Action in Diabetes: Evaluation of Cardiovascular Outcome Results; PIONEER 6 - Peptide Innovation for Early Diabetes Treatment 6; REWIND - Researching Cardiovascular Events with a Weekly Incretin in Diabetes; SELECT - Semaglutide Effects on Cardiovascular Outcomes in People with Overweight or Obesity; SOUL - Semaglutide Cardiovascular Outcomes Trial; SUSTAIN-6 - Evaluate Cardiovascular and Other Long-term Outcomes with Semaglutide in Subjects with Type 2 Diabetes; T2D – Type 2 Diabetes Mellitus; N/A - not available

# **Supplementary Table 5: Extended Study Characteristics and Endpoint definitions**

| **Study Name (year)** | **First Author** | **Total Participants (n)** | **Intervention** | **Indication** | **Inclusion criteria** | **Exclusion criteria** |
| --- | --- | --- | --- | --- | --- | --- |
| **AMPLITUDE-O (2021)** | Gerstein | 4076 | Efpeglenatide | DM and high CV risk | Adults with T2D and HbA1c > 7% and history of CV disease or age ≥50 years (if male) or ≥ 55 years (if female) and CKD and at least one additional CV risk factor | Gastroparesis, uncontrolled reflux, prolonged nausea or vomiting, severe retinal disease, pancreatitis, or use of GLP-1RA or DPP-4i within the previous 3 months |
| **ELIXA (2015)** | Pfeffer | 6068 | Lixisenatide | DM and high CV risk | Age ≥ 30 years with T2D and ACS event within 180 days before screening | PCI in prior 15 days, CABG for the qualifying event, planned coronary revascularization procedure within 90 days after screening, eGFR <30 mL/min per 1.73 m^2^, HbA1c < 5.5% or > 11.0%, or an inability to provide written informed consent. |
| **EXSCEL (2017)** | Holman | 14752 | Exenatide | DM and high CV risk | Adults with T2D | History of ≥ 2 severe hypoglycemia episodes during the preceding 12 months, end-stage CKD or eGFR <30 mL/min per 1.73 m^2^, personal or family history of medullary thyroid carcinoma or MEN2, a baseline calcitonin level ≥ 40 ng/l, or previous GLP-1RA use |
| **FLOW (2024)** | Perkovic | 3553 | Semaglutide | DM and high CV risk | T2D and HbA1c ≤10% with high-risk CKD and receiving a stable maximal labelled dose (or the maximal dose without unacceptable side effects) of ACEI or ARB | Hypersensitivity to trial products, pregnancy or breastfeeding, congenital kidney disease, MI, stroke/TIA or PCI in prior 60 days, planned revascularization, prior cancer in the past 5 years |
| **FREEDOM-CVO (2022)** | Ruff | 4156 | Exenatide | DM and high CV risk | Age ≥ 40 years with T2D and established cardiovascular disease or at high CV risk | Prior treatment with DPP4i or GLP-1RA or SGLT2i, current use of rapid acting insulin or immunosuppression or agents affecting GI motility, history of pancreatitis or thyroid cancer or thyroid nodule, raised TSH not investigated, NYHA III/IV heart failure, malignancy, eGFR <50 mL/min per 1.73 m^2^ |
| **Harmony Outcomes (2018)** | Hernandez | 9463 | Albiglutide | DM and high CV risk | Age ≥ 40 years with T2D and HbA1c ≥ 7·0% and established CV disease | Severe gastroparesis, eGFR < 30 mL/min per 1.73 m^2^, previous pancreatitis or substantial risk factors for pancreatitis, a personal or family history of medullary carcinoma of the thyroid or MEN2, history of pancreatic neuroendocrine tumour, current GLP-1RA use |
| **LEADER (2016)** | Marso | 9340 | Liraglutide | DM and high CV risk | Age ≥ 50 years with T2D and HbA1c ≥7% and at least one CV condition or age ≥ 60 years with at least one CV risk factor | T1D, use of GLP-1RA, DPP-4i, pramlintide or rapid-acting insulin, familial or personal history of MEN2 or medullary thyroid cancer; ACS or stroke/TIA in 14 days prior to screening and randomization |
| **PIONEER 6 (2019)** | Husain | 3183 | Semaglutide | DM and high CV risk | Age ≥50 years with T2D and established CV disease or CKD, or age ≥ 60 years and CV risk factors only | GLP-1RA, DPP4i or pramlintide within 90 days before screening; NYHA IV HF; planned revascularizarion of a coronary, carotid, or peripheral artery; MI, stroke, or hospitalization for unstable angina or TIA within 60 days before screening; long-term or intermittent hemodialysis or peritoneal dialysis, or severe renal impairment and proliferative retinopathy or maculopathy resulting in active treatment |
| **REWIND (2019)** | Gerstein | 9901 | Dulaglutide | DM and high CV risk | Age ≥ 50 years with T2D and HbA1c ≤ 9·5% on stable doses of up to two oral glucose-lowering drugs with or without basal insulin therapy and BMI ≥ 23 kg/m^2^ and CV disease if aged ≥ 55 years and at least 2 of tobacco use, dyslipidaemia, hypertension or abdominal obesity if aged ≥ 60 years | Cancer in the previous 5 years, severe hypoglycaemia in the previous year, life expectancy less than 1 year, a coronary or cerebrovascular event within the previous 2 months, and plans for revascularisation, eGFR <15mL/min per 1.73 m^2^ |
| **SELECT**  **(2023)** | Lincoff | 17604 | Semaglutide | Obesity and high CV risk | Age ≥45 years with BMI ≥ 27 kg/m^2^, and established CV disease | DM, HbA1c ≥ 6.5%, use of glucose-lowering medication or GLP-1RA within prior 90 days, NYHA IV HF, end-stage kidney disease or dialysis, CV or neurological event in previous 60 days, planned revascularisation of a coronary, carotid or peripheral artery |
| **SOUL (2025)** | McGuire | 9650 | Semaglutide | DM and high CV risk | Age ≥50 and T2D with HbA1c 6.5 - 10.0% and at least one of: coronary artery disease, cerebrovascular disease, symptomatic peripheral artery disease, or CKD | End-stage CKD or receipt of long-term kidney-replacement therapy |
| **SUSTAIN 6**  **(2016)** | Marso | 3297 | Semaglutide | DM and high CV risk | Age ≥50 years with T2D and established CV disease, chronic HF (NYHA II or III), or CKD stage 3 or higher or age ≥60 years with at least one CV risk factor | DPP4i use within 30 days; use of GLP-1RA or insulin other than basal or premixed within 90 days; ACS or stroke/TIA within 90 days; planned revascularization of a coronary, carotid, or peripheral artery; long-term dialysis. |

ACEI – angiotensin converting enzyme inhibitor; ACS – acute coronary syndrome; AMPLITUDE-O - Effect of Efpeglenatide on Cardiovascular Outcomes; ARB – angiotensin receptor blocker; BMI – Body Mass Index; CABG – coronary artery bypass grafting; CKD – chronic kidney disease; CV – cardiovascular; DM – Diabetes Mellitus; DPP4i – dipeptidylpeptidase-4 inhibitor; eGFR – estimated glomerular filtration rate; ELIXA - Evaluation of Lixisenatide in Acute Coronary Syndrome; EXSCEL - Exenatide Study of Cardiovascular Event Lowering; FLOW - Evaluate Renal Function with Semaglutide Once Weekly; FREEDOM-CVO - Subcutaneous infusion of exenatide and cardiovascular outcomes in type 2 diabetes; GI – gastrointestinal; GLP-1RA – Glucagon-Like Receptor 1 agonist; Harmony Outcomes - Albiglutide and cardiovascular outcomes in patients with type 2 diabetes and cardiovascular disease; HbA1c – glycosylated haemoglobin; HF – Heart Failure; LEADER - Liraglutide Effect and Action in Diabetes: Evaluation of Cardiovascular Outcome Results; LVEF – Left Ventricular Ejection Fraction; MEN2 – multiple endocrine neoplasia type 2; NYHA – New York Heart Association; PCI – percutaneous coronary intervention; PIONEER 6 - Peptide Innovation for Early Diabetes Treatment 6; REWIND - Researching Cardiovascular Events with a Weekly Incretin in Diabetes; SELECT - Semaglutide Effects on Cardiovascular Outcomes in People with Overweight or Obesity; SOUL - Semaglutide Cardiovascular Outcomes Trial; SUSTAIN-6 - Evaluate Cardiovascular and Other Long-term Outcomes with Semaglutide in Subjects with Type 2 Diabetes; T1D – Type 1 Diabetes Mellitus; T2D – Type 2 Diabetes Mellitus; TIA – transient ischaemic attack

**Supplementary Table 6: Meta-regression**

| Factors | Risk ratio (95% CI) | P-value |
| --- | --- | --- |
| Age | 0.98 (0.95-1.02) | 0.311 |
| BMI | 0.98 (0.89-1.07) | 0.605 |
| Median follow up (months) | 1.00 (1.00-1.00) | 0.797 |
| Total patients (n) | 1.00 (1.00-1.00) | 0.911 |

BMI, body mass index

# **Supplementary Table 7: Adverse Events per study**

| **Study Name** | **Acute pancreatitis intervention arm (n)** | **Acute pancreatitis control arm (n)** | **Acute renal failure intervention arm (n)** | **Acute renal failure control arm (n)** | **Severe hypoglycaemia intervention arm (n)** | **Severe hypoglycaemia control arm (n)** | **Cancer intervention arm (n)** | **Cancer**  **control arm (n)** |
| --- | --- | --- | --- | --- | --- | --- | --- | --- |
| **AMPLITUDE-O** | 11 | 7 | 88 | 39 | 24 | 13 | 72 | 37 |
| **ELIXA** | N/A | N/A | N/A | N/A | N/A | N/A | N/A | N/A |
| **EXSCEL** | 26 | 22 | N/A | N/A | 247 | 219 | 355 | 361 |
| **FLOW** | 10 | 7 | 172 | 182 | 37 | 37 | 120 | 104 |
| **FREEDOM-CVO** | 8 | 2 | 39 | 24 | 8 | 4 | N/A | N/A |
| **Harmony Outcomes** | 10 | 7 | 279 | 316 | 31 | 55 | 87 | 112 |
| **LEADER** | 18 | 23 | N/A | N/A | 114 | 153 | 296 | 279 |
| **PIONEER 6** | 1 | 3 | 32 | 37 | 23 | 13 | 41 | 48 |
| **REWIND** | 23 | 13 | 84 | 93 | 64 | 74 | 351 | 348 |
| **SELECT** | 17 | 24 | 171 | 200 | N/A | N/A | 422 | 418 |
| **SOUL** | 18 | 21 | 148 | 168 | 76 | 84 | 332 | 294 |
| **SUSTAIN 6** | 9 | 12 | 65 | 69 | 369 | 350 | 66 | 70 |

AMPLITUDE-O - Effect of Efpeglenatide on Cardiovascular Outcomes; ELIXA - Evaluation of Lixisenatide in Acute Coronary Syndrome; EXSCEL - Exenatide Study of Cardiovascular Event Lowering; FLOW - Evaluate Renal Function with Semaglutide Once Weekly; FREEDOM-CVO - Subcutaneous infusion of exenatide and cardiovascular outcomes in type 2 diabetes; Harmony Outcomes - Albiglutide and cardiovascular outcomes in patients with type 2 diabetes and cardiovascular disease; LEADER - Liraglutide Effect and Action in Diabetes: Evaluation of Cardiovascular Outcome Results; N/A - not available; PIONEER 6 - Peptide Innovation for Early Diabetes Treatment 6; REWIND - Researching Cardiovascular Events with a Weekly Incretin in Diabetes; SELECT - Semaglutide Effects on Cardiovascular Outcomes in People with Overweight or Obesity; SOUL - Semaglutide Cardiovascular Outcomes Trial; SUSTAIN-6 - Evaluate Cardiovascular and Other Long-term Outcomes with Semaglutide in Subjects with Type 2 Diabetes

# **Supplementary Figure 1: The PRISMA flowchart of studies included for systematic review and meta-analysis.**

**Identification of studies via databases and registers**

Records identified from*:

Databases:

Medline (n = 897)

Embase (n = 1035)

Cochrane (n = 347)

Total (n = 2279)

Records removed *before screening*:

Duplicate records removed

(n = 312)

**Identification**

Records screened

(n = 1967)

Records excluded**

(n = 1882)

Reports sought for retrieval

(n = 85)

Reports not retrieved

(n = 0)

**Screening**

Reports excluded:

Other Heart Failure Outcome (n = 29)

Secondary Analysis (n = 27)

Review/Systematic Review/Comment/Editorial/ Recommendations/Guideline/Case report/Case Series (n = 17)

Duplicates (n = 1)

Non-English study (n=1)

Reports assessed for eligibility

(n = 85)

**Included**

Studies included in review

(n = 10)

Studies included in quantitative synthesis

(n = 10)

Additional records identified through backward/forward citation and expert consultation

(n=0)

# **Supplementary Figure 2: Risk of Bias per study**


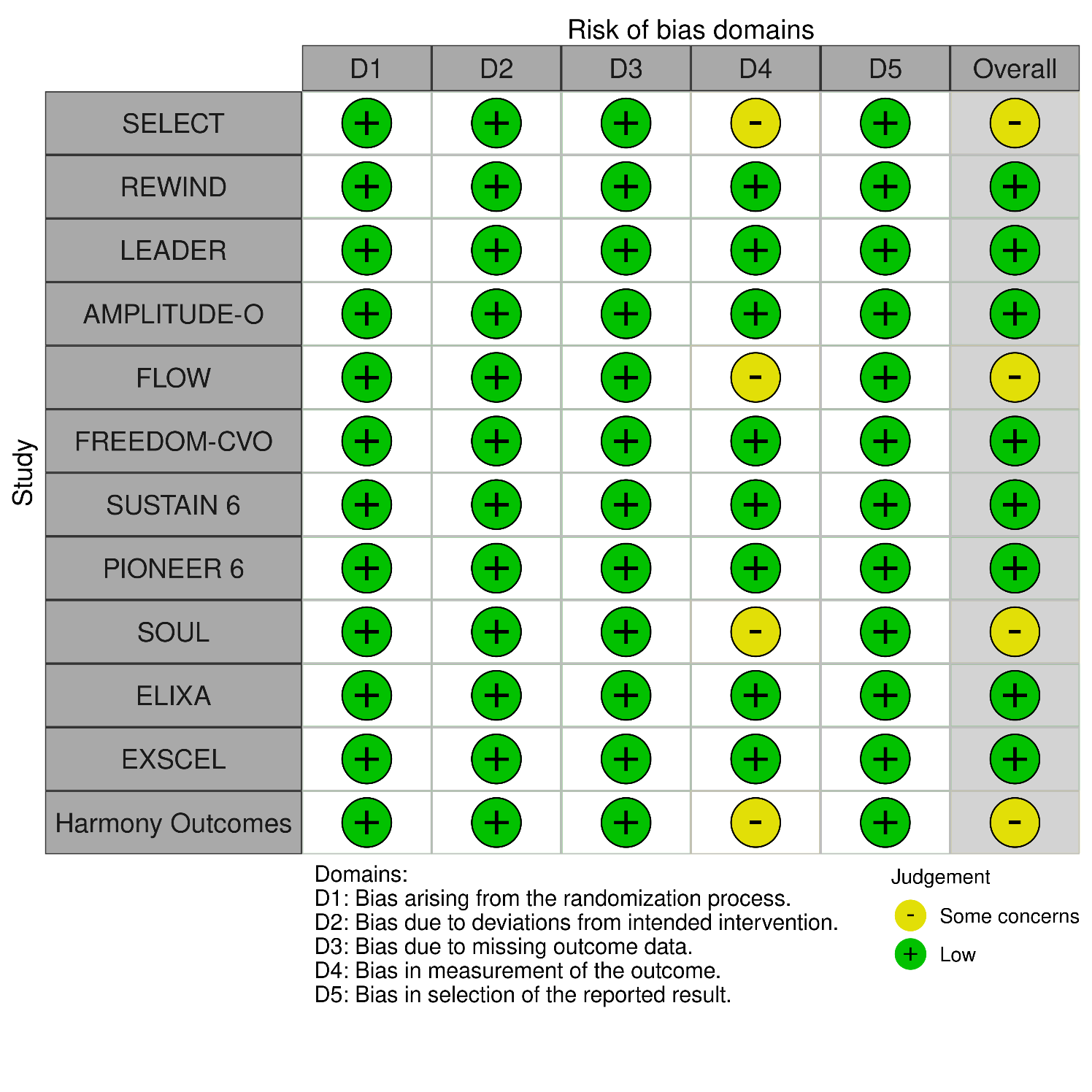


AMPLITUDE-O - Effect of Efpeglenatide on Cardiovascular Outcomes; ELIXA - Evaluation of Lixisenatide in Acute Coronary Syndrome; EXSCEL - Exenatide Study of Cardiovascular Event Lowering; FLOW - Evaluate Renal Function with Semaglutide Once Weekly; FREEDOM-CVO - Subcutaneous infusion of exenatide and cardiovascular outcomes in type 2 diabetes; Harmony Outcomes - Albiglutide and cardiovascular outcomes in patients with type 2 diabetes and cardiovascular disease; LEADER - Liraglutide Effect and Action in Diabetes: Evaluation of Cardiovascular Outcome Results; PIONEER 6 - Peptide Innovation for Early Diabetes Treatment 6; REWIND - Researching Cardiovascular Events with a Weekly Incretin in Diabetes; SELECT - Semaglutide Effects on Cardiovascular Outcomes in People with Overweight or Obesity; SOUL - Semaglutide Cardiovascular Outcomes Trial; SUSTAIN-6 - Evaluate Cardiovascular and Other Long-term Outcomes with Semaglutide in Subjects with Type 2 Diabetes

# **Supplementary Figure 3: Summary plot of Risk of Bias**


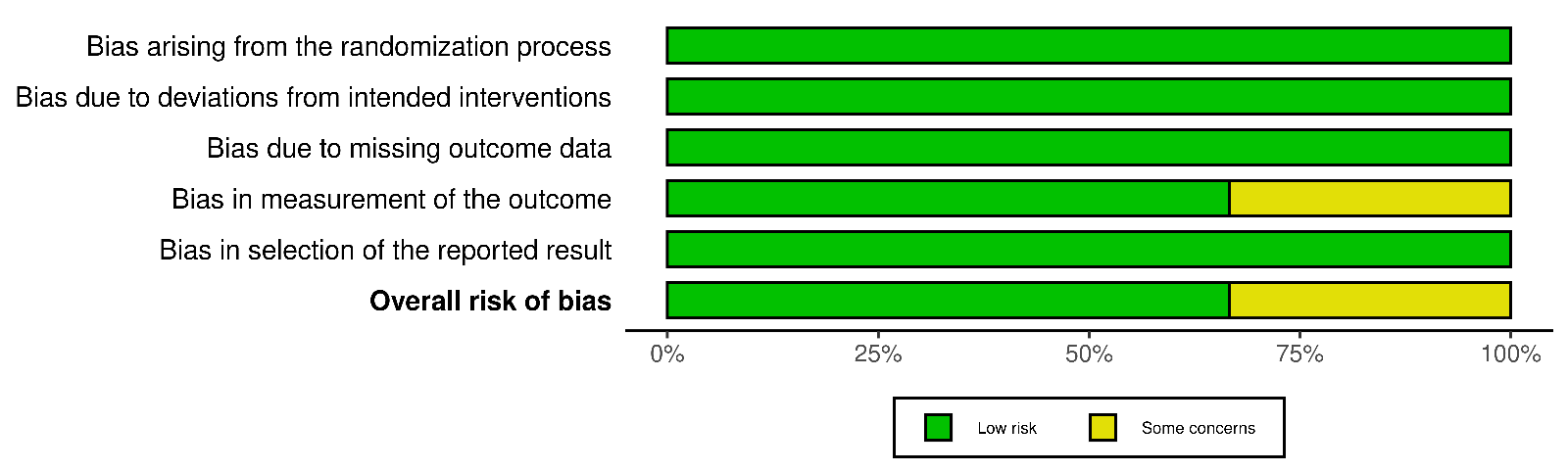


# **Supplementary Figure 4: Summary plot of semaglutide in patients without baseline heart failure across the FLOW and SELECT trials, stratified by indication**


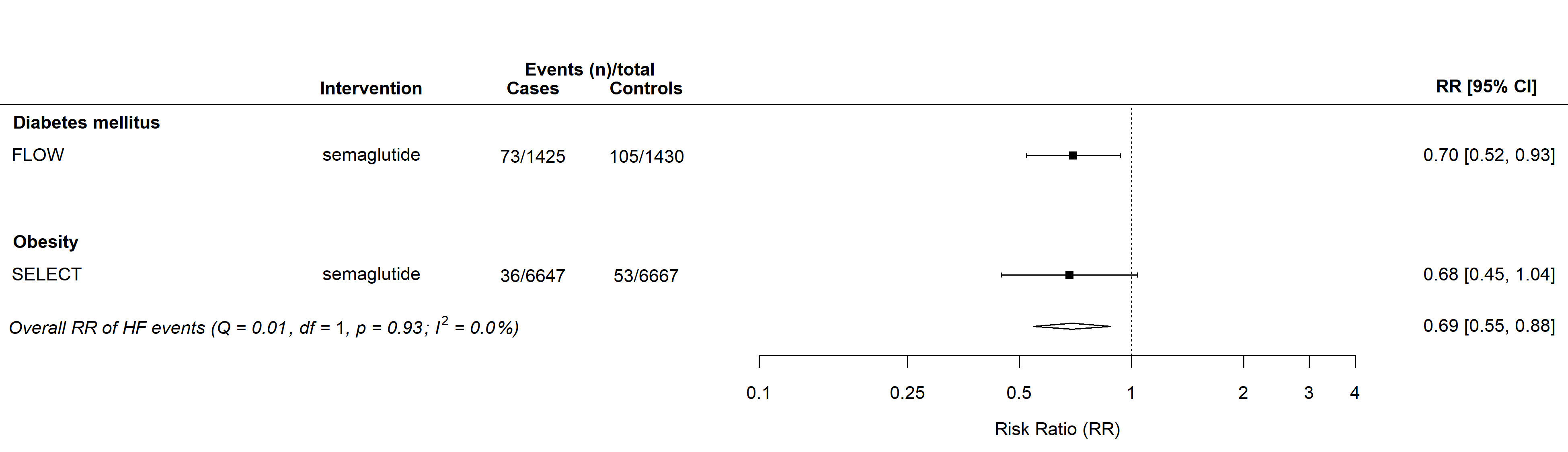


FLOW - Evaluate Renal Function with Semaglutide Once Weekly; HF – heart failure; SELECT - Semaglutide Effects on Cardiovascular Outcomes in People with Overweight or Obesity

# **Supplementary Figure 5: Summary plot of GLP-1 receptor agonists for heart failure events excluding the FLOW trial, stratified by indication**


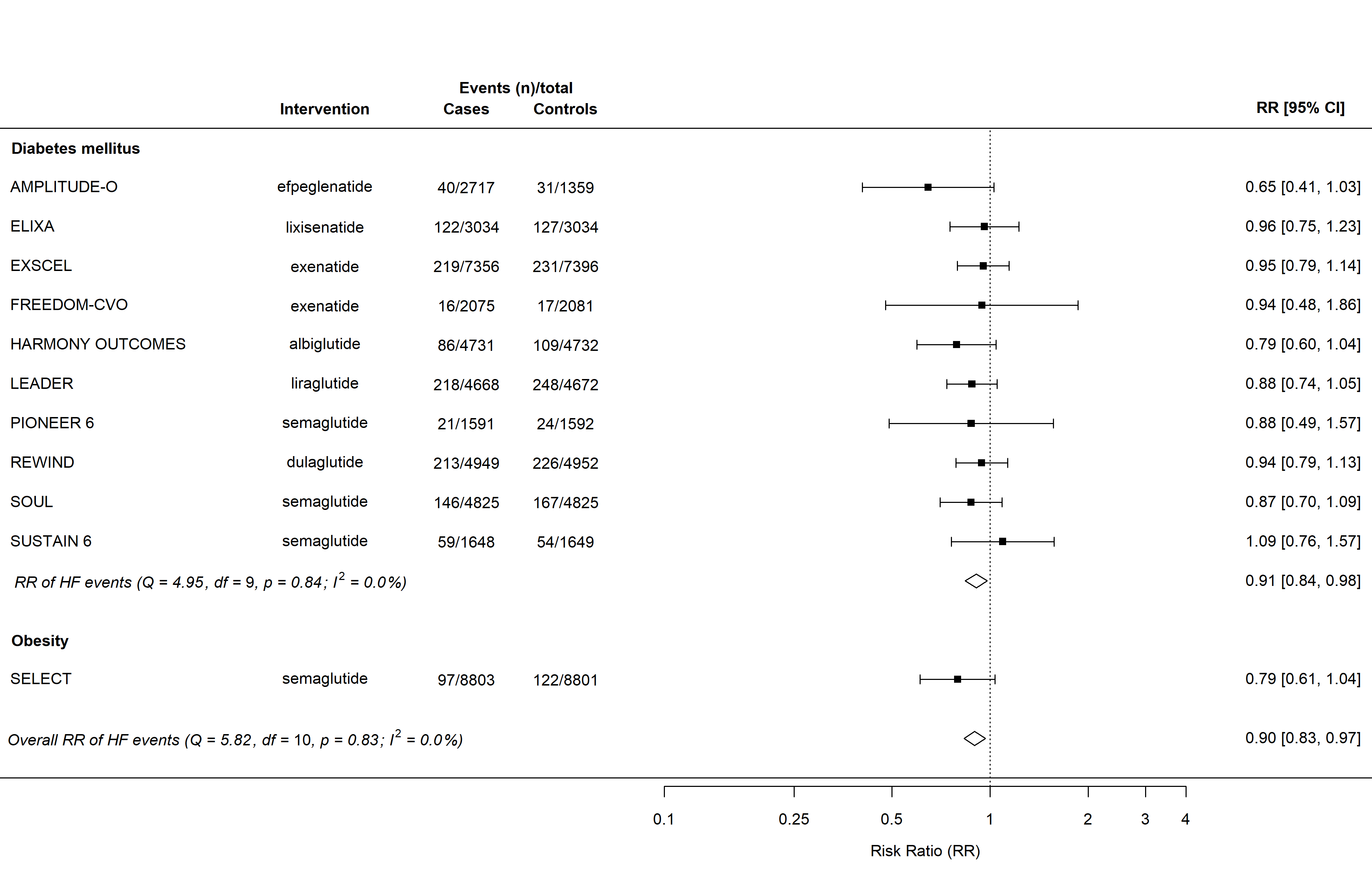


AMPLITUDE-O - Effect of Efpeglenatide on Cardiovascular Outcomes; ELIXA - Evaluation of Lixisenatide in Acute Coronary Syndrome; EXSCEL - Exenatide Study of Cardiovascular Event Lowering; FREEDOM-CVO - Subcutaneous infusion of exenatide and cardiovascular outcomes in type 2 diabetes; Harmony Outcomes - Albiglutide and cardiovascular outcomes in patients with type 2 diabetes and cardiovascular disease; HF – heart failure; LEADER - Liraglutide Effect and Action in Diabetes: Evaluation of Cardiovascular Outcome Results; PIONEER 6 - Peptide Innovation for Early Diabetes Treatment 6; REWIND - Researching Cardiovascular Events with a Weekly Incretin in Diabetes; SELECT - Semaglutide Effects on Cardiovascular Outcomes in People with Overweight or Obesity; SOUL - Semaglutide Cardiovascular Outcomes Trial; SUSTAIN-6 - Evaluate Cardiovascular and Other Long-term Outcomes with Semaglutide in Subjects with Type 2 Diabetes

# **Supplementary Figure 6: Summary plot of GLP-1 receptor agonist safety outcomes; acute pancreatitis**


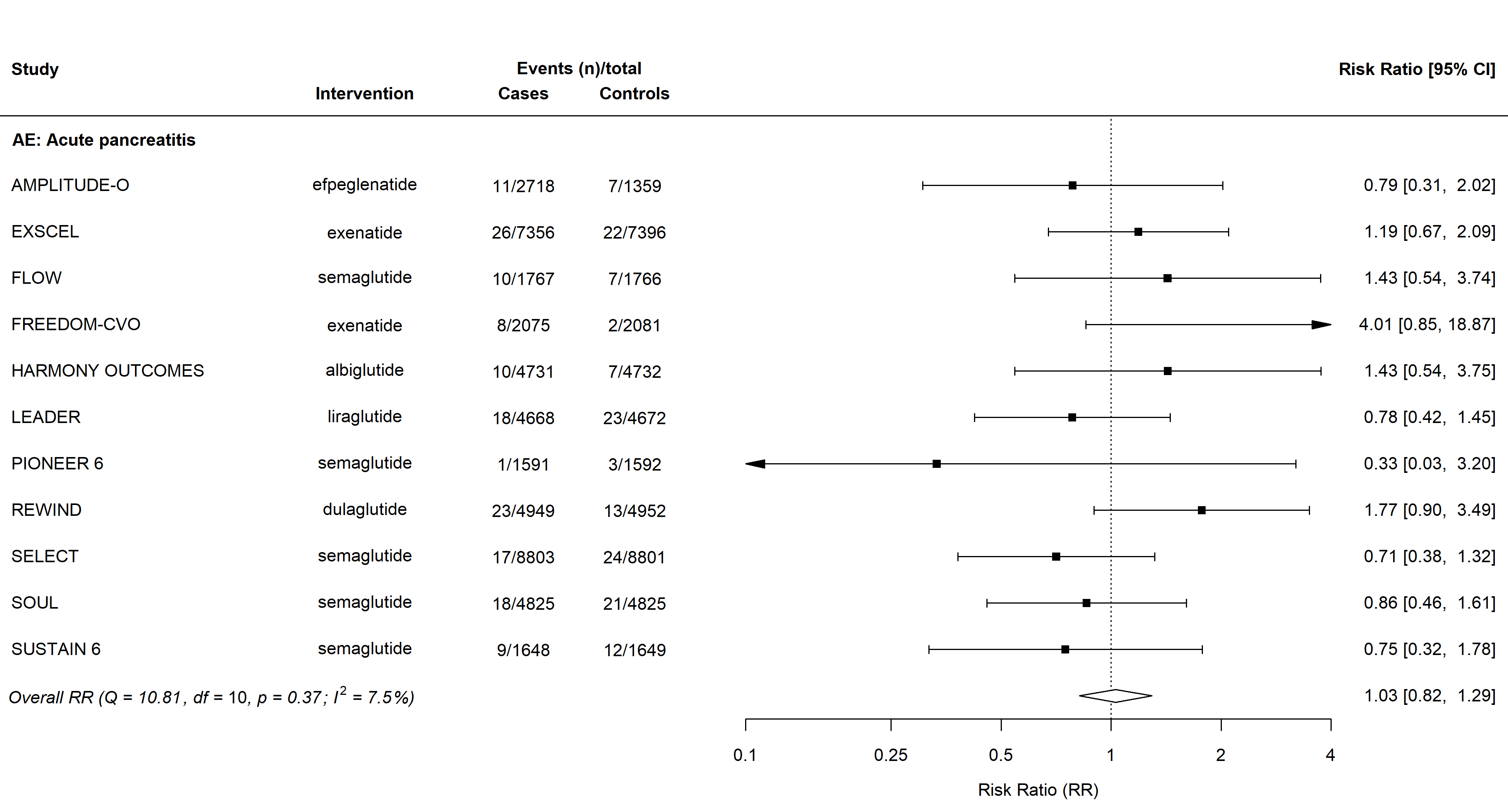
AMPLITUDE-O - Effect of Efpeglenatide on Cardiovascular Outcomes; EXSCEL - Exenatide Study of Cardiovascular Event Lowering; FLOW - Evaluate Renal Function with Semaglutide Once Weekly; FREEDOM-CVO - Subcutaneous infusion of exenatide and cardiovascular outcomes in type 2 diabetes; Harmony Outcomes - Albiglutide and cardiovascular outcomes in patients with type 2 diabetes and cardiovascular disease; LEADER - Liraglutide Effect and Action in Diabetes: Evaluation of Cardiovascular Outcome Results; PIONEER 6 - Peptide Innovation for Early Diabetes Treatment 6; REWIND - Researching Cardiovascular Events with a Weekly Incretin in Diabetes; SELECT - Semaglutide Effects on Cardiovascular Outcomes in People with Overweight or Obesity; SOUL - Semaglutide Cardiovascular Outcomes Trial; SUSTAIN-6 - Evaluate Cardiovascular and Other Long-term Outcomes with Semaglutide in Subjects with Type 2 Diabetes

# **Supplementary Figure 7: Summary plot of GLP-1 receptor agonist safety outcomes; severe hypoglycaemia**


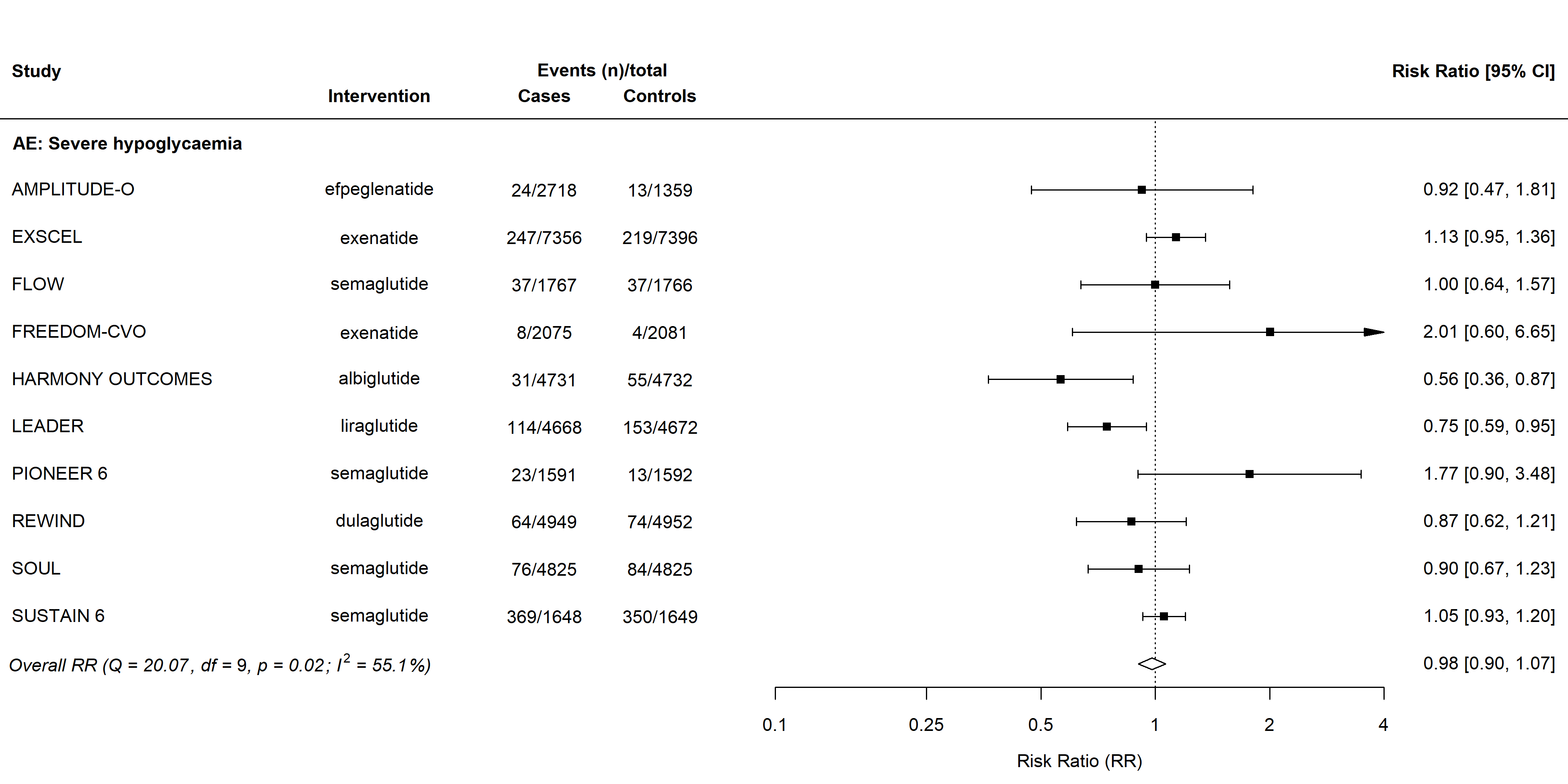


AMPLITUDE-O - Effect of Efpeglenatide on Cardiovascular Outcomes; EXSCEL - Exenatide Study of Cardiovascular Event Lowering; FLOW - Evaluate Renal Function with Semaglutide Once Weekly; FREEDOM-CVO - Subcutaneous infusion of exenatide and cardiovascular outcomes in type 2 diabetes; Harmony Outcomes - Albiglutide and cardiovascular outcomes in patients with type 2 diabetes and cardiovascular disease; LEADER - Liraglutide Effect and Action in Diabetes: Evaluation of Cardiovascular Outcome Results; PIONEER 6 - Peptide Innovation for Early Diabetes Treatment 6; REWIND - Researching Cardiovascular Events with a Weekly Incretin in Diabetes; SOUL - Semaglutide Cardiovascular Outcomes Trial; SUSTAIN-6 - Evaluate Cardiovascular and Other Long-term Outcomes with Semaglutide in Subjects with Type 2 Diabetes

# **Supplementary Figure 8: Summary plot of GLP-1 receptor agonist safety outcomes; acute renal failure**


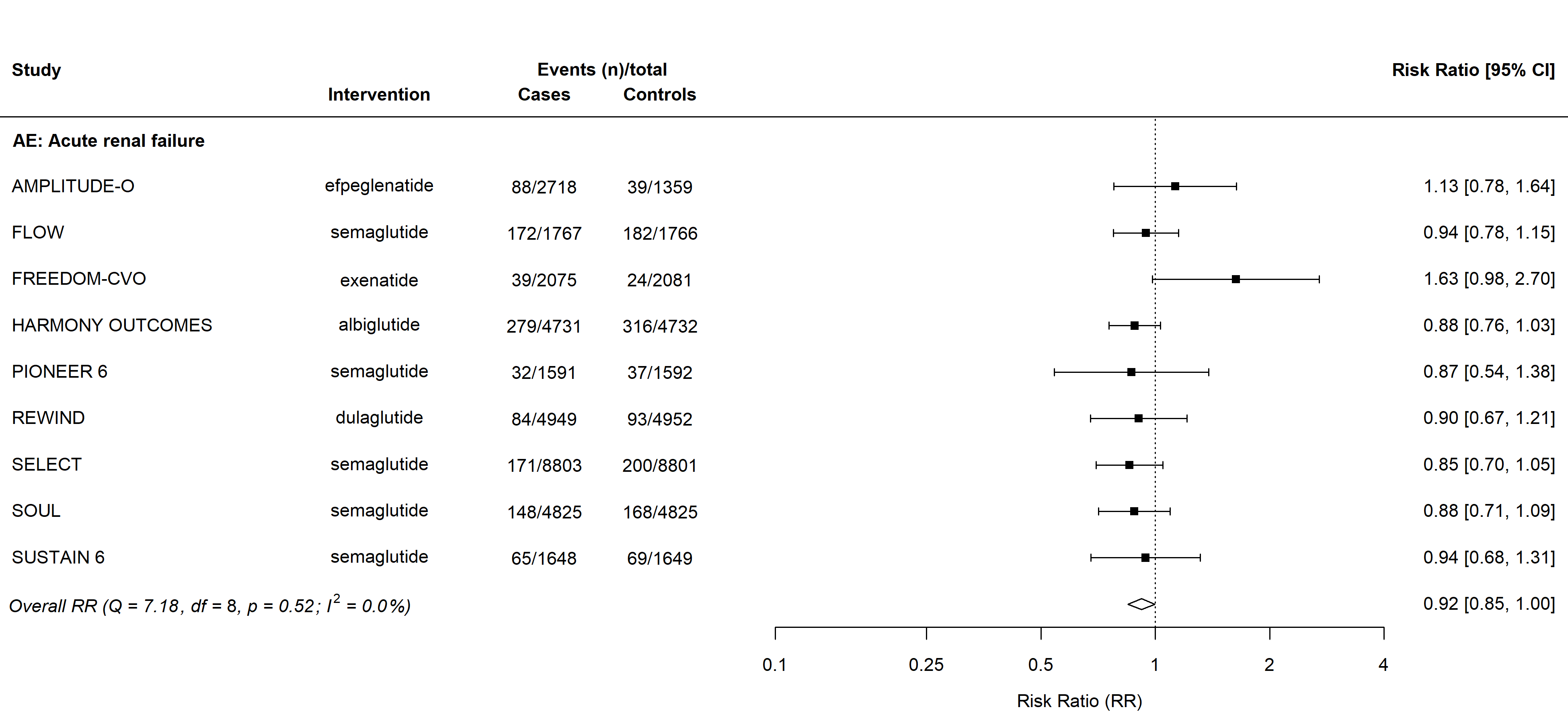
AMPLITUDE-O - Effect of Efpeglenatide on Cardiovascular Outcomes; FLOW - Evaluate Renal Function with Semaglutide Once Weekly; FREEDOM-CVO - Subcutaneous infusion of exenatide and cardiovascular outcomes in type 2 diabetes; Harmony Outcomes - Albiglutide and cardiovascular outcomes in patients with type 2 diabetes and cardiovascular disease; PIONEER 6 - Peptide Innovation for Early Diabetes Treatment 6; REWIND - Researching Cardiovascular Events with a Weekly Incretin in Diabetes; SELECT - Semaglutide Effects on Cardiovascular Outcomes in People with Overweight or Obesity; SOUL - Semaglutide Cardiovascular Outcomes Trial; SUSTAIN-6 - Evaluate Cardiovascular and Other Long-term Outcomes with Semaglutide in Subjects with Type 2 Diabetes

# **Supplementary Figure 9: Summary plot of GLP-1 receptor agonist safety outcomes; cancer**


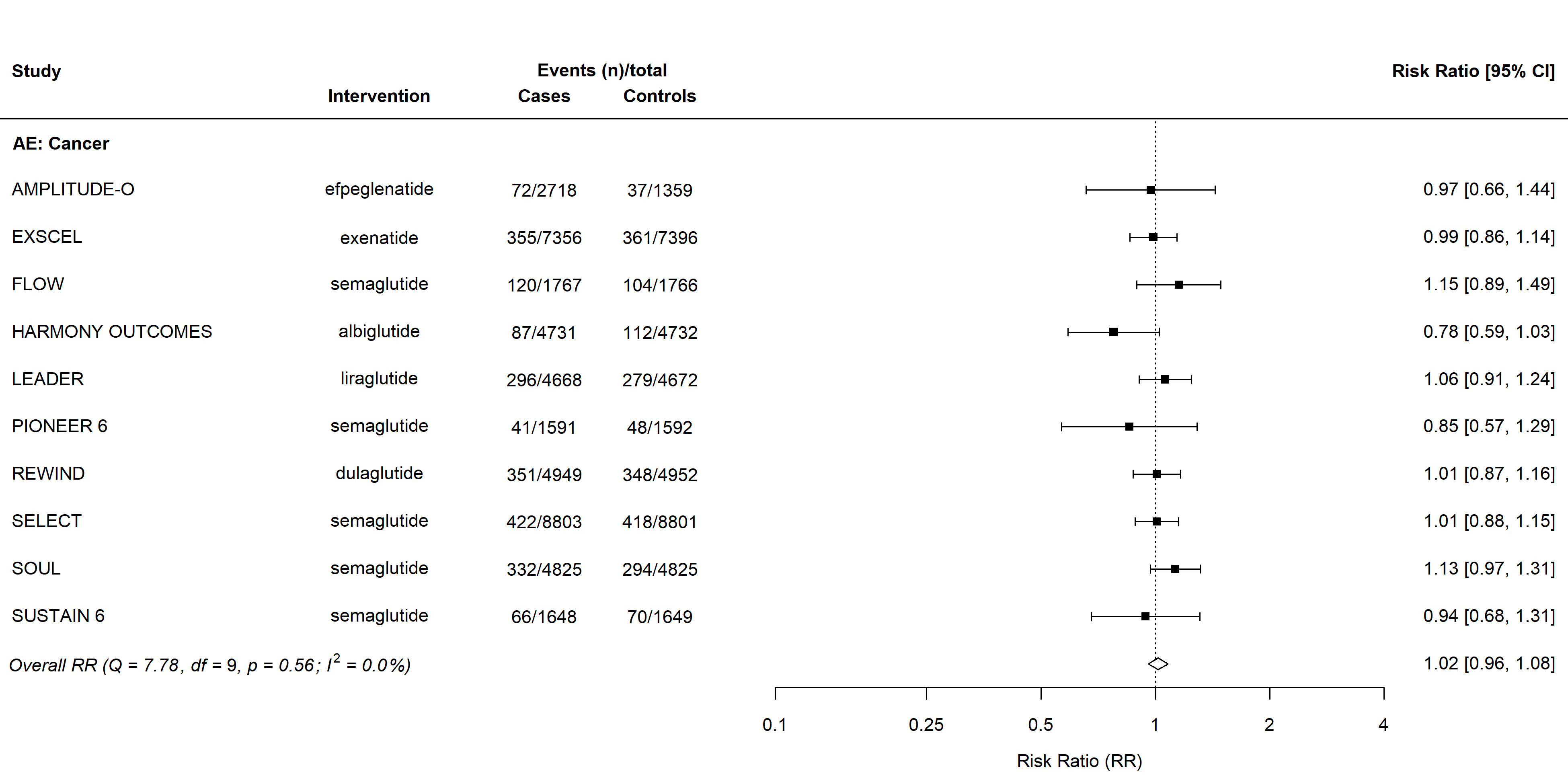


AMPLITUDE-O - Effect of Efpeglenatide on Cardiovascular Outcomes; EXSCEL - Exenatide Study of Cardiovascular Event Lowering; FLOW - Evaluate Renal Function with Semaglutide Once Weekly; Harmony Outcomes - Albiglutide and cardiovascular outcomes in patients with type 2 diabetes and cardiovascular disease; LEADER - Liraglutide Effect and Action in Diabetes: Evaluation of Cardiovascular Outcome Results; PIONEER 6 - Peptide Innovation for Early Diabetes Treatment 6; REWIND - Researching Cardiovascular Events with a Weekly Incretin in Diabetes; SELECT - Semaglutide Effects on Cardiovascular Outcomes in People with Overweight or Obesity; SOUL - Semaglutide Cardiovascular Outcomes Trial; SUSTAIN-6 - Evaluate Cardiovascular and Other Long-term Outcomes with Semaglutide in Subjects with Type 2 Diabetes

# **Supplementary Figure 10: Summary plot of Semaglutide specific safety outcomes; acute pancreatitis**


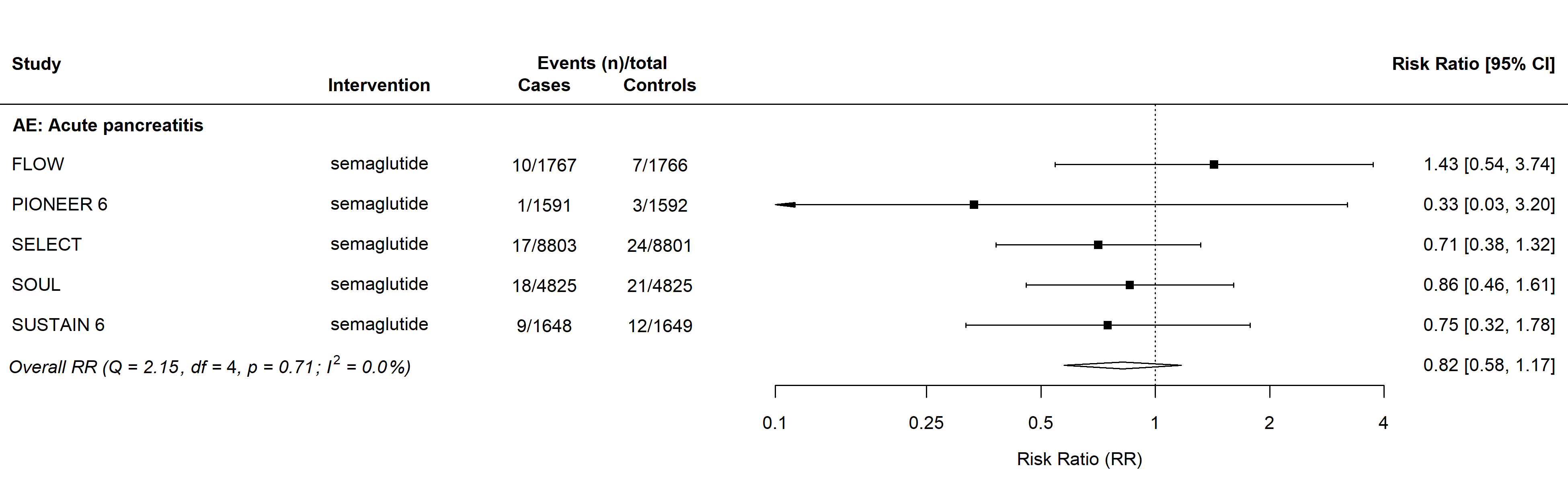


FLOW - Evaluate Renal Function with Semaglutide Once Weekly; PIONEER 6 - Peptide Innovation for Early Diabetes Treatment 6; SELECT - Semaglutide Effects on Cardiovascular Outcomes in People with Overweight or Obesity; SOUL - Semaglutide Cardiovascular Outcomes Trial; SUSTAIN-6 - Evaluate Cardiovascular and Other Long-term Outcomes with Semaglutide in Subjects with Type 2 Diabetes

# **Supplementary Figure 11: Summary plot of Semaglutide specific safety outcomes; severe hypoglycaemia**


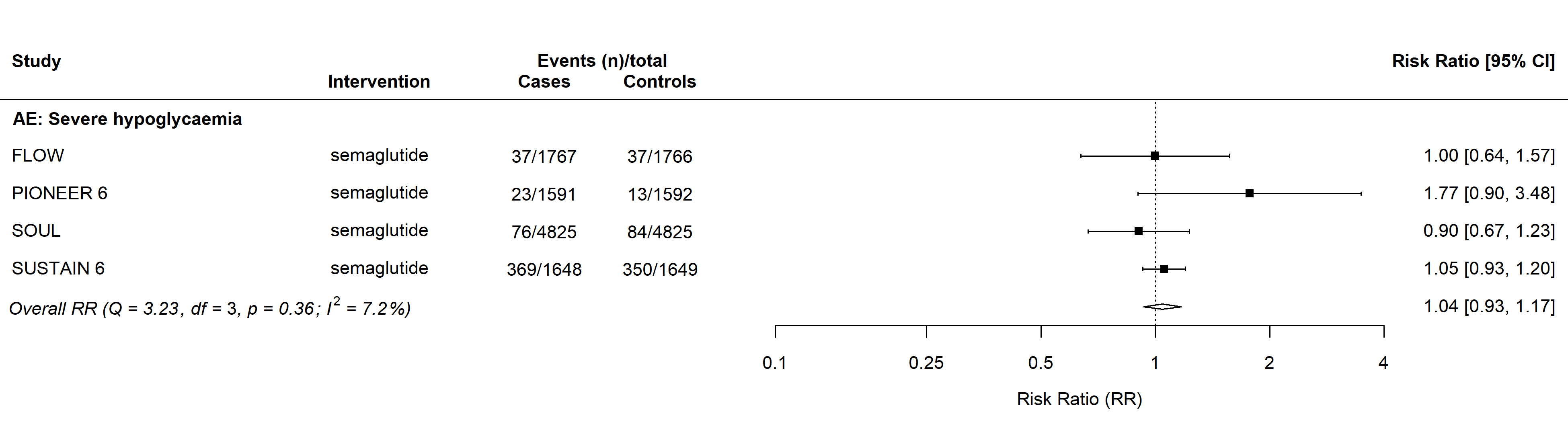


FLOW - Evaluate Renal Function with Semaglutide Once Weekly; PIONEER 6 - Peptide Innovation for Early Diabetes Treatment 6; SOUL - Semaglutide Cardiovascular Outcomes Trial; SUSTAIN-6 - Evaluate Cardiovascular and Other Long-term Outcomes with Semaglutide in Subjects with Type 2 Diabetes;

# **Supplementary Figure 12: Summary plot of Semaglutide specific safety outcomes; acute renal failure**


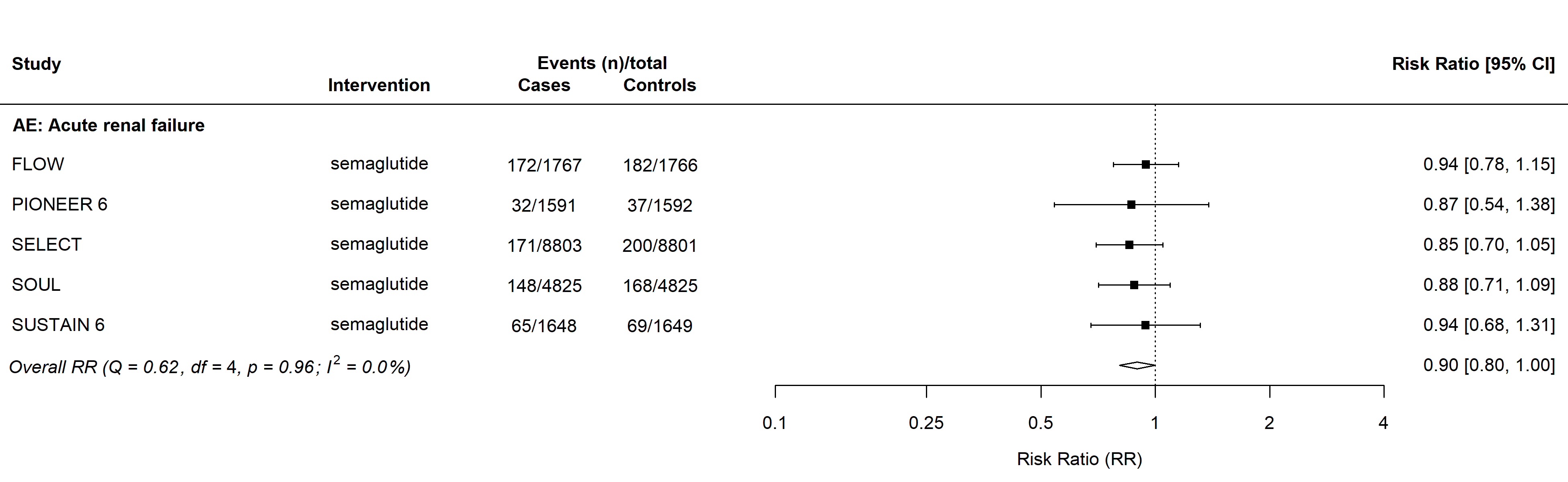
FLOW - Evaluate Renal Function with Semaglutide Once Weekly; PIONEER 6 - Peptide Innovation for Early Diabetes Treatment 6; SELECT - Semaglutide Effects on Cardiovascular Outcomes in People with Overweight or Obesity; SOUL - Semaglutide Cardiovascular Outcomes Trial; SUSTAIN-6 - Evaluate Cardiovascular and Other Long-term Outcomes with Semaglutide in Subjects with Type 2 Diabetes

# **Supplementary Figure 13: Summary plot of Semaglutide specific safety outcomes; cancer**


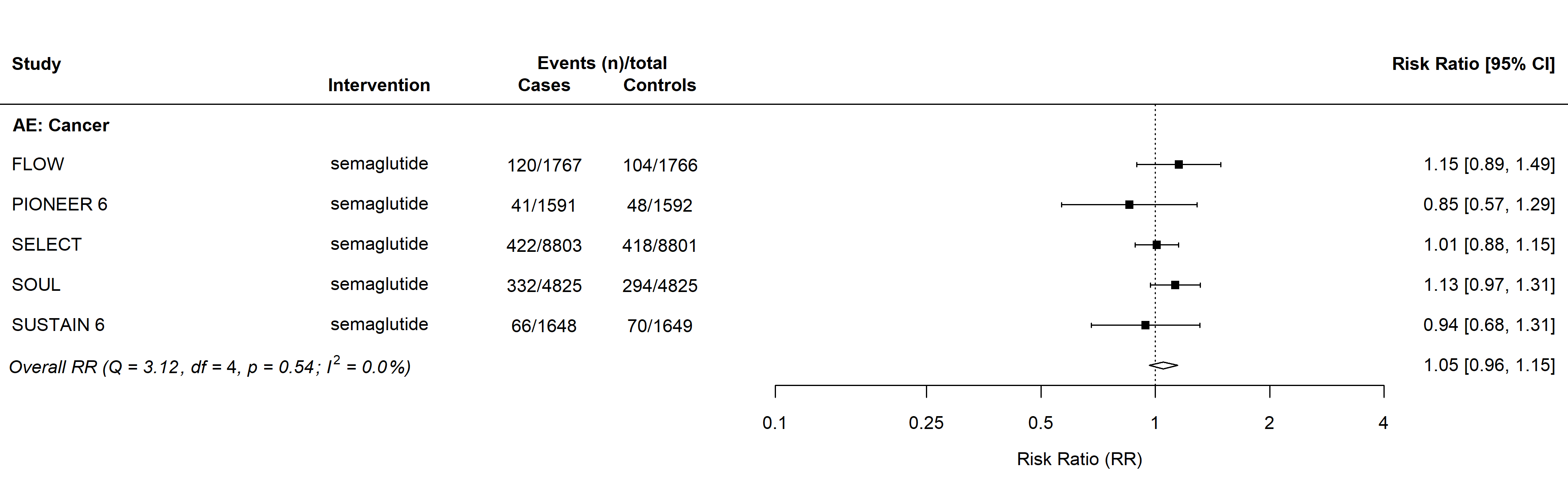
FLOW - Evaluate Renal Function with Semaglutide Once Weekly; PIONEER 6 - Peptide Innovation for Early Diabetes Treatment 6; SELECT - Semaglutide Effects on Cardiovascular Outcomes in People with Overweight or Obesity; SOUL - Semaglutide Cardiovascular Outcomes Trial; SUSTAIN-6 - Evaluate Cardiovascular and Other Long-term Outcomes with Semaglutide in Subjects with Type 2 Diabetes

| **Section and Topic** | **Item #** | **Checklist item** | **Reported (Yes/No)** |
| --- | --- | --- | --- |
| **TITLE** | | |  |
| Title | 1 | Identify the report as a systematic review. | Yes |
| **BACKGROUND** | | |  |
| Objectives | 2 | Provide an explicit statement of the main objective(s) or question(s) the review addresses. | Yes |
| **METHODS** | | |  |
| Eligibility criteria | 3 | Specify the inclusion and exclusion criteria for the review. | Yes |
| Information sources | 4 | Specify the information sources (e.g. databases, registers) used to identify studies and the date when each was last searched. | Yes |
| Risk of bias | 5 | Specify the methods used to assess risk of bias in the included studies. | No, is detailed in the manuscript |
| Synthesis of results | 6 | Specify the methods used to present and synthesise results. | Yes |
| **RESULTS** | | |  |
| Included studies | 7 | Give the total number of included studies and participants and summarise relevant characteristics of studies. | Yes |
| Synthesis of results | 8 | Present results for main outcomes, preferably indicating the number of included studies and participants for each. If meta-analysis was done, report the summary estimate and confidence/credible interval. If comparing groups, indicate the direction of the effect (i.e. which group is favoured). | Yes |
| **DISCUSSION** | | |  |
| Limitations of evidence | 9 | Provide a brief summary of the limitations of the evidence included in the review (e.g. study risk of bias, inconsistency and imprecision). | No, is detailed in the manuscript |
| Interpretation | 10 | Provide a general interpretation of the results and important implications. | Yes |
| **OTHER** | | |  |
| Funding | 11 | Specify the primary source of funding for the review. | Detailed on page 2-3 |
| Registration | 12 | Provide the register name and registration number. | Detailed on page 4 |

*From:*  Page MJ, McKenzie JE, Bossuyt PM, Boutron I, Hoffmann TC, Mulrow CD, et al. The PRISMA 2020 statement: an updated guideline for reporting systematic reviews. BMJ 2021;372:n71. doi: 10.1136/bmj.n71. This work is licensed under CC BY 4.0. To view a copy of this license, visit <https://creativecommons.org/licenses/by/4.0/>

| **Section and Topic** | **Item #** | **Checklist item** | **Location where item is reported** |
| --- | --- | --- | --- |
| **TITLE** | | |  |
| Title | 1 | Identify the report as a systematic review. | Page 1 |
| **ABSTRACT** | | |  |
| Abstract | 2 | See the PRISMA 2020 for Abstracts checklist. | See separate form |
| **INTRODUCTION** | | |  |
| Rationale | 3 | Describe the rationale for the review in the context of existing knowledge. | Page 7 |
| Objectives | 4 | Provide an explicit statement of the objective(s) or question(s) the review addresses. | Page 7 |
| **METHODS** | | |  |
| Eligibility criteria | 5 | Specify the inclusion and exclusion criteria for the review and how studies were grouped for the syntheses. | Page 8 |
| Information sources | 6 | Specify all databases, registers, websites, organisations, reference lists and other sources searched or consulted to identify studies. Specify the date when each source was last searched or consulted. | Page 8 |
| Search strategy | 7 | Present the full search strategies for all databases, registers and websites, including any filters and limits used. | Supplementary Material pages 3-6 |
| Selection process | 8 | Specify the methods used to decide whether a study met the inclusion criteria of the review, including how many reviewers screened each record and each report retrieved, whether they worked independently, and if applicable, details of automation tools used in the process. | Pages 8-9 |
| Data collection process | 9 | Specify the methods used to collect data from reports, including how many reviewers collected data from each report, whether they worked independently, any processes for obtaining or confirming data from study investigators, and if applicable, details of automation tools used in the process. | Pages 8-9 |
| Data items | 10a | List and define all outcomes for which data were sought. Specify whether all results that were compatible with each outcome domain in each study were sought (e.g. for all measures, time points, analyses), and if not, the methods used to decide which results to collect. | Pages 8-9 |
|  | 10b | List and define all other variables for which data were sought (e.g. participant and intervention characteristics, funding sources). Describe any assumptions made about any missing or unclear information. | Pages 8-9 |
| Study risk of bias assessment | 11 | Specify the methods used to assess risk of bias in the included studies, including details of the tool(s) used, how many reviewers assessed each study and whether they worked independently, and if applicable, details of automation tools used in the process. | Page 9 |
| Effect measures | 12 | Specify for each outcome the effect measure(s) (e.g. risk ratio, mean difference) used in the synthesis or presentation of results. | Page 9 |
| Synthesis methods | 13a | Describe the processes used to decide which studies were eligible for each synthesis (e.g. tabulating the study intervention characteristics and comparing against the planned groups for each synthesis (item #5)). | Page 9 |
|  | 13b | Describe any methods required to prepare the data for presentation or synthesis, such as handling of missing summary statistics, or data conversions. | Pages 9-10 |
|  | 13c | Describe any methods used to tabulate or visually display results of individual studies and syntheses. | Pages 9-10 |
|  | 13d | Describe any methods used to synthesize results and provide a rationale for the choice(s). If meta-analysis was performed, describe the model(s), method(s) to identify the presence and extent of statistical heterogeneity, and software package(s) used. | Pages 9-10 |
|  | 13e | Describe any methods used to explore possible causes of heterogeneity among study results (e.g. subgroup analysis, meta-regression). | Page 9 |
|  | 13f | Describe any sensitivity analyses conducted to assess robustness of the synthesized results. | Pages 9-10 |
| Reporting bias assessment | 14 | Describe any methods used to assess risk of bias due to missing results in a synthesis (arising from reporting biases). | Page 10 |
| Certainty assessment | 15 | Describe any methods used to assess certainty (or confidence) in the body of evidence for an outcome. | Page 9 |
| **RESULTS** | | |  |
| Study selection | 16a | Describe the results of the search and selection process, from the number of records identified in the search to the number of studies included in the review, ideally using a flow diagram. | Page 11 and Supplementary Figure 1 |
|  | 16b | Cite studies that might appear to meet the inclusion criteria, but which were excluded, and explain why they were excluded. | N/A |
| Study characteristics | 17 | Cite each included study and present its characteristics. | Pages 11-12 and Supplementary Tables 1-7 (Pg 8-16) |
| Risk of bias in studies | 18 | Present assessments of risk of bias for each included study. | Page 12 and Supplementary Figures 2-3 (Pg 18-19) |
| Results of individual studies | 19 | For all outcomes, present, for each study: (a) summary statistics for each group (where appropriate) and (b) an effect estimate and its precision (e.g. confidence/credible interval), ideally using structured tables or plots. | Pages 11-14, Figures 1-3 and Supplementary Figures 4-13 |
| Results of syntheses | 20a | For each synthesis, briefly summarise the characteristics and risk of bias among contributing studies. | Pages 11-14 |
|  | 20b | Present results of all statistical syntheses conducted. If meta-analysis was done, present for each the summary estimate and its precision (e.g. confidence/credible interval) and measures of statistical heterogeneity. If comparing groups, describe the direction of the effect. | Pages 11-14, Figures 1-3, Supplementary Table 6 and Supplementary Figures 4-13 |
|  | 20c | Present results of all investigations of possible causes of heterogeneity among study results. | Figures 1-3 |
|  | 20d | Present results of all sensitivity analyses conducted to assess the robustness of the synthesized results. | Page 14 |
| Reporting biases | 21 | Present assessments of risk of bias due to missing results (arising from reporting biases) for each synthesis assessed. | Supplementary Figures 2-3 (Pg 18-19) |
| Certainty of evidence | 22 | Present assessments of certainty (or confidence) in the body of evidence for each outcome assessed. | Pages 11-14, Figures 1-3, Supplementary table 6 and Supplementary Figures 4-13 |
| **DISCUSSION** | | |  |
| Discussion | 23a | Provide a general interpretation of the results in the context of other evidence. | Page 15-18 |
|  | 23b | Discuss any limitations of the evidence included in the review. | Page 18 |
|  | 23c | Discuss any limitations of the review processes used. | Page 18 |
|  | 23d | Discuss implications of the results for practice, policy, and future research. | Page 16-18 |
| **OTHER INFORMATION** | | |  |
| Registration and protocol | 24a | Provide registration information for the review, including register name and registration number, or state that the review was not registered. | Page 8 |
|  | 24b | Indicate where the review protocol can be accessed, or state that a protocol was not prepared. | Page 8 |
|  | 24c | Describe and explain any amendments to information provided at registration or in the protocol. | N/A |
| Support | 25 | Describe sources of financial or non-financial support for the review, and the role of the funders or sponsors in the review. | Pages 20-21 |
| Competing interests | 26 | Declare any competing interests of review authors. | Pages 20-21 |
| Availability of data, code and other materials | 27 | Report which of the following are publicly available and where they can be found: template data collection forms; data extracted from included studies; data used for all analyses; analytic code; any other materials used in the review. | Page 21 |

*From:*  Page MJ, McKenzie JE, Bossuyt PM, Boutron I, Hoffmann TC, Mulrow CD, et al. The PRISMA 2020 statement: an updated guideline for reporting systematic reviews. BMJ 2021;372:n71. doi: 10.1136/bmj.n71. This work is licensed under CC BY 4.0. To view a copy of this license, visit <https://creativecommons.org/licenses/by/4.0/>
